# A systems pharmacology approach based on oncogenic signalling pathways to determine the mechanisms of action of natural products in breast cancer from transcriptome data

**DOI:** 10.1186/s12906-021-03340-z

**Published:** 2021-06-30

**Authors:** Regan Odongo, Asuman Demiroglu-Zergeroglu, Tunahan Çakır

**Affiliations:** 1 Department of Bioengineering, Gebze Technical University, Gebze, Kocaeli, Turkey; 2 Department of Molecular Biology and Genetics, Gebze Technical University, Gebze, Kocaeli, Turkey

**Keywords:** Systems pharmacology, Transcriptomics, Plant-based drugs, Breast cancer, Oncogenic signalling pathways

## Abstract

**Background:**

Narrow spectrum of action through limited molecular targets and unforeseen drug-related toxicities have been the main reasons for drug failures at the phase I clinical trials in complex diseases. Most plant-derived compounds with medicinal values possess poly-pharmacologic properties with overall good tolerability, and, thus, are appropriate in the management of complex diseases, especially cancers. However, methodological limitations impede attempts to catalogue targeted processes and infer systemic mechanisms of action. While most of the current understanding of these compounds is based on reductive methods, it is increasingly becoming clear that holistic techniques, leveraging current improvements in omic data collection and bioinformatics methods, are better suited for elucidating their systemic effects. Thus, we developed and implemented an integrative systems biology pipeline to study these compounds and reveal their mechanism of actions on breast cancer cell lines.

**Methods:**

Transcriptome data from compound-treated breast cancer cell lines, representing triple negative (TN), luminal A (ER+) and HER2+ tumour types, were mapped on human protein interactome to construct targeted subnetworks. The subnetworks were analysed for enriched oncogenic signalling pathways. Pathway redundancy was reduced by constructing pathway-pathway interaction networks, and the sets of overlapping genes were subsequently used to infer pathway crosstalk. The resulting filtered pathways were mapped on oncogenesis processes to evaluate their anti-carcinogenic effectiveness, and thus putative mechanisms of action.

**Results:**

The signalling pathways regulated by Actein, Withaferin A, Indole-3-Carbinol and Compound Kushen, which are extensively researched compounds, were shown to be projected on a set of oncogenesis processes at the transcriptomic level in different breast cancer subtypes. The enrichment of well-known tumour driving genes indicate that these compounds indirectly dysregulate cancer driving pathways in the subnetworks.

**Conclusion:**

The proposed framework infers the mechanisms of action of potential drug candidates from their enriched protein interaction subnetworks and oncogenic signalling pathways. It also provides a systematic approach for evaluating such compounds in polygenic complex diseases. In addition, the plant-based compounds used here show poly-pharmacologic mechanism of action by targeting subnetworks enriched with cancer driving genes. This network perspective supports the need for a systemic drug-target evaluation for lead compounds prior to efficacy experiments.

**Supplementary Information:**

The online version contains supplementary material available at 10.1186/s12906-021-03340-z.

## Background

While reductionism-based approaches generated most of the drugs and drug targets known today, drug-human interactions are rather complex and the mechanism of action of most pharmacologically effective drugs results from the perturbation of cellular networks [1]. Thus, a phenotypic change following a drug treatment is the result of multiple dysregulation cascades covering various biomolecular interactions, which can be traced using the omics framework [1, 2]. Within this framework, several studies have utilized transcriptomic data to generate novel hypotheses regarding the mechanism of drug actions from drug-driven transcriptome perturbations in various diseases. However, new perspectives on the development and use of computational frameworks are needed in order to translate information generated from such data into a clear mechanism of action for drugs in the context of systems biology [2].

Most cancers are driven by multiple genetic mutations and epigenetic dysregulations [3, 4] interconnected by different molecular players. Breast cancer is the most prevalent form of cancer in women [5]. Distinct tumour subtypes have been defined for this cancer, and inter/intra-group subtle genetic variations are known to exist [6]. Owing to the understanding of the existence of somatic gene mutations that aggregate in a few signalling and regulatory pathways [7], a number of small molecule targeted therapies have been developed for breast cancer in the last decade. However, treatment success rates above 40% are yet to be recorded [5]. A plausible explanation to this is the inherent growth-promoting oncogenic signalling pathways that crosstalk with other activating pathways thereby making it difficult to therapeutically stop tumour growth. Unfortunately, most of the currently available drugs and their evaluation methods are based on their ability to inhibit a single gene target in these pathways, which is insufficient in breast cancer and other polygenic diseases since these diseases often harbour multiple gene mutations. This explicitly points to the need for a multi-targeted systemic therapeutic approach, as well as a systematic framework for the evaluation of candidate drugs.

Existing methods for drug discovery and drug target identification are highly efficient in elucidating the mechanism of action of anti-microbial drugs. However, studies have consistently demonstrated that this simple framework is inefficient in addressing drug action in complex and multi-factorial disease systems. In such systems, limiting drug research to targeting single disease biomarkers is one of the main causes of drug failures in clinical trials [1, 2, 8]. Drug induced reprogramming of cellular responses is directed through metabolic reactions, which are regulated by signalling pathways enormously enriched in protein-protein interactions. Thus, studying drug effects on cellular pathways provides a holistic approach for the identification of molecular targets of drug candidates. Given the increased preference by tumours for only a handful number of such pathways, a sound anti-carcinogenic effect can thus be deduced by evaluating their activity upon treatment. A recent study evaluating oncogenesis related pathways based on gene profiling in various cancers [9] provides a foundation for systemically evaluating the therapeutic relevance of drug-responsive pathways upon treatment in various tumours. Thus, we postulated that a network and pathway-based approach would provide an accurate picture of systemic effects of candidate drugs in complex polygenic diseases.

Experimental evidences from separate studies on the use of plant-based drugs in cancer cells have strongly suggested a multi-targeting therapeutic strategy. In fact, ancient civilizations relied on plant-based compounds due to their relatively low systemic toxicities and ability to simultaneously treat multiple closely-related disease conditions [10]. Actein, a triterpene glycoside isolated from *Cimicifuga foetida* medicinal plant [11], Withaferin A, a steroidal lactone from Withania sominfera [12] plant, Compound Kushen Injection, a Chinese herb prepared from *Sophora flavescens* and *Heterosimilax chinensis* medicinal plants [13], and indole-3-carbinol, a plant phytohormone from cruciferous vegetables [14], are amongst the most widely studied and documented plant-derived compounds in breast cancer. Justifiably, current systems biology analyses through differential gene expression enumerations have confirmed similar observations [15–17]. Yet, despite this, no attempt has been made to integrate transcriptome-level response to these drugs with molecular interaction networks to systematically evaluate the mechanism of action of these compounds. Emboldened by the idea that condition-specific co-regulated and co-expressed proteins tend to converge on well-defined biological pathways, we hypothesised that genes targeted by plant-based compounds exert a pleiotropic effect on multiple oncogenic pathways that modulate the response to treatment. To discern the mechanisms reflected by these perturbed subnetworks, we catalogued all the molecular players involved and used them in enrichment analysis.

Network biology is a holistic approach in systems biology to understand biological systems, where biomolecules and their binary interactions are projected onto a graph to depict molecular relationships [18]. Nowadays, concurrent integration of experimentally-derived omics data with a priori interaction data is a common approach in systems biology to obtain context-specific subnetworks [19]. To this end, a number of computational tools have been proposed by different groups to map and construct subnetworks from transcriptome data [20] and applied to several diseases, including breast cancer [21], hepatocellular carcinoma [22, 23], liver fibrosis [24] and neurodegenerative diseases [25, 26]. However, the use of this powerful concept to systematically detail the mechanism of action of lead compounds is still underexplored as most current studies still often diverge to the exclusive receptor-ligand binding paradigm to discover drug targets, thereby limiting the amount of biological information that can be gained from such compounds.

In this study, we developed a data-centric computational framework to determine the mechanism of action of plant derived natural products on breast cancer cell lines. In this approach, we mapped compound-treated breast cancer transcriptome data (actein [11], compound kushen injection (CKI) [13], indole-3-carbinol [14] and Withaferin A [12]) on protein interactome and constructed the underlying subnetworks. We used network topology metrics to validate the relatedness of these subnetworks with human breast cancer disease cases. Next, we performed pathway enrichment analysis to extract enriched signalling pathways, which were then used to define the mechanisms of action of each drug by (i) constructing pathway gene-similarity interactomes and (ii) mapping these pathways on carcinogenesis processes from literature sources. Overall, we showed that these compounds possess poly-pharmacologic properties and target oncogenic signalling pathways, which can be mapped on carcinogenesis processes of therapeutic importance. Notably, we found that multiple compound-perturbed oncogenic signalling pathways work together to control same cancer-driving carcinogenesis processes.

## Methods

**Fig. 1:**
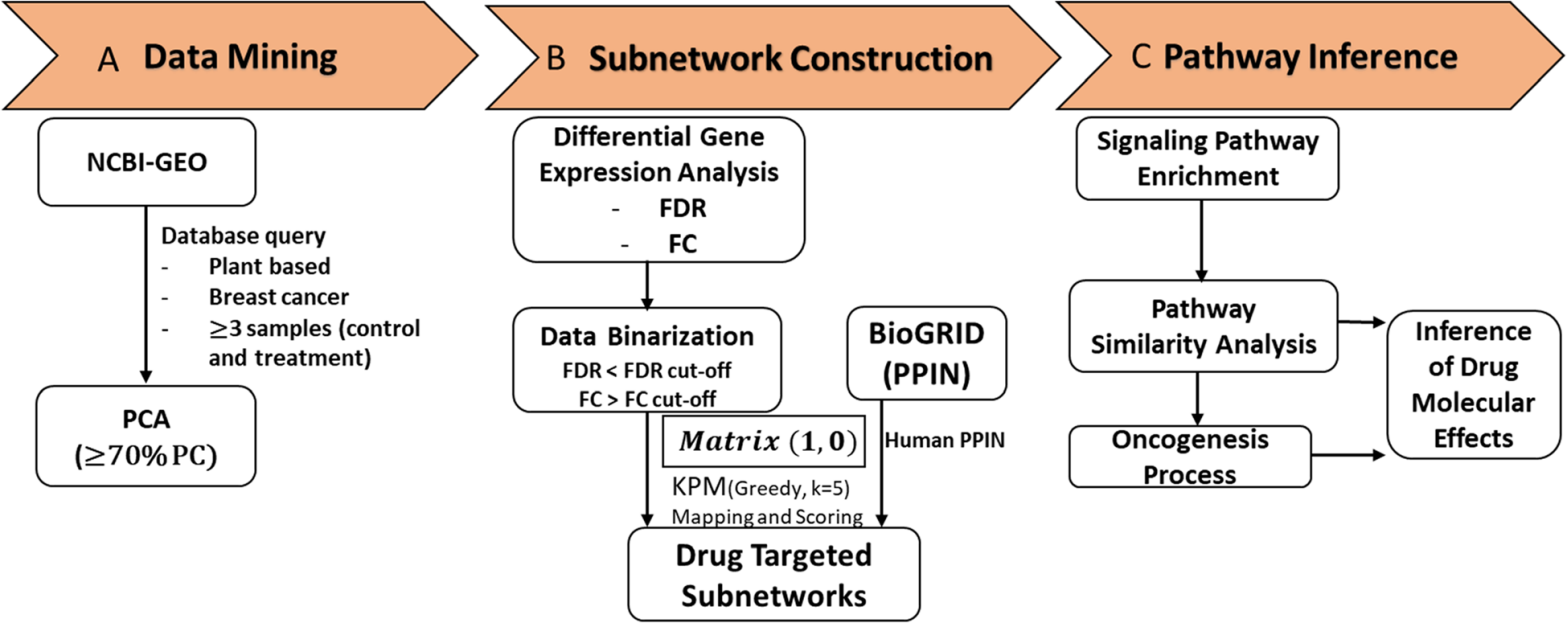
Computational analysis workflow applied in this study. The approach is centred on three main analysis sections: data mining, subnetwork discovery and pathway inference. PCA: Principal component analysis, FDR: False discovery rate, FC: Fold change, KPM: KeyPathwayMiner, PPIN: Protein-protein interaction network

The computational analysis steps followed in this study are summarized in Fig. 1.

### Data acquisition

We used a structured query statement to interrogate and download gene expression datasets for the breast cancer cell lines treated with withaferin A (GSE53049) [12], actein (GSE7848) [11], CKI (GSE78512) [13] and indole-3-carbinol (GSE55897) [14] from the NCBI GEO repository. We selected these four plant-based compounds among others since the corresponding datasets had at least 3 control and 3 treatment groups, and there was a distinct separation between the control and treatment groups (tested using the unsupervised dimension reduction method, principal component analysis).

### Data processing and differential gene expression analysis

The expression datasets included microarray expression profiles and RNA-seq counts and, therefore, platform specific protocols were followed. For the microarray derived datasets (withaferin A, actein and indole-3-carbinol), probeset mapping was performed by choosing the probe with the maximum average expression value among multiple probesets of a gene. For RNA-seq data (CKI), we selected only those genes with above zero counts in at least two samples in either control or treatment group. Overall, we log2 normalized all the pre-processed datasets. Subsequently, we used LIMMA [27] package in R to identify differentially expressed genes between the treated versus control (untreated) groups. We used Benjamini-Hochberg *p*-value correction to control for false discovery rates (FDR). Fold change and FDR cut-offs were simultaneously used to select differentially expressed genes.

### Active subnetwork scoring and construction using KeyPathwayMiner

The challenge of discovering most-connected drug specific subnetworks in the human protein-protein interaction network was solved using KeyPathwayMiner (KPM) [28], one of the tools reported to have a high performance among subnetwork discovery methods [20]. In this approach, given a priori protein-protein interaction network (PPIN), we were interested in a maximally connected clique based on a significance score. Hence, we treat this problem as an optimization problem with two main constraints: (i) the maximum allowable non-differentially expressed genes, and (ii) the significance cut-off. In this work, we used the Cytoscape (v3.7.1) based KPM (v5.0.1) plugin.

In our analysis, we made a few modifications to the input data and constraints as we describe next. We applied a uniform fold-change cut-off of 2 and a varied FDR cut-off of 5 × 10^−3^ (for indole-3-carbinol and withaferin A) or 1 × 10^−2^ (for actein and CKI) to identify differentially expressed genes. Thus, our approach is strict; with the intention of reducing the rate of false positives and retaining only important features. These two cut-offs were used to assign binary values to all the genes in a dataset. Specifically, we used ‘1’ to denote differentially expressed genes based on our criteria, and ‘0’ for other genes. In the subnetwork construction, significantly changed and physically interacting proteins are used. These interconnected proteins essentially denote drug-targeted cellular pathways. We allowed a maximum of 5 non-differentially expressed genes in each subnetwork solution, a parameter available in KPM. For the priori human PPIN, we used BioGRID [29] (release 3.5.173; 25th March, 2019) containing 22,435 proteins and 478,529 interactions.

### Subnetwork analysis and prospective validation of high centrality genes

Using CytoNCA (v2.1.6) [30] Cytoscape plugin, we analysed two network topological features to identify the major genes in the subnetworks: degree (connectivity) and betweenness centrality. Degree centrality measures the number of interactions made by a given gene while betweenness centrality measures the importance of a given gene in the network by computing the relative number of shortest paths passing through a given gene [31].Next, we used the TCGA breast cancer RNA-Seq data to investigate the prognostic characteristics of the top 5 (based on high degree and betweenness centrality) identified genes. Specifically, we used the online tool KM-Express [32] to determine the effect of the identified genes on overall survival and their association with samples from normal, primary and metastatic cases. For the overall survival, the tool uses the median gene expression across all samples and a hazard ratio to infer statistical significance based on log-rank *p*-value and returns a Kaplan-Meier curve detailing the relationship between the expression of a given gene and overall survival as captured in the TCGA [33] clinical data. A p-value cut-off of 0.05 was used in this study.

### Pathway enrichment analysis

We used enrichR [34] package in R to perform pathway enrichment analysis for the respective subnetwork nodes (genes). It uses pathway definitions from Kyoto Encyclopaedia of Genes and Genomes (KEGG) [35], WikiPathway [36], Reactome [37] and Gene Ontology Biological Process (GO-BP) [38] databases, among others. We limited our results to the enriched pathways with an FDR cut-off of 0.05 and containing the terms: ‘signal’, ‘apoptosis’, and ‘cell cycle’. Also, those pathways with less than 3 associated genes were removed at this step.

### Construction of pathway-pathway interaction network

Oncogenic signalling pathways do not function in isolation but are known to crosstalk with each other while redirecting cellular processes. Construction of pathway interaction networks has been previously applied to visually elaborate the pathway-pathway interrelationships and infer associated biological phenomenon [39, 40]. On the other hand, since pathway enrichment from enrichR was based on multiple pathway databases, redundant pathways were inevitable in the enrichment results. Therefore, pathway-pathway similarity can also be used to identify redundant pathways. One approach to computationally enumerate such relationships is to evaluate the degree of pathway-pathway overlap based on gene similarities in any given two pathways. We used the Jaccard index, which is a measure of the similarity between a pair of sets. Here, given two pathways, *P*_*i*_ and *P*_*j*_, with enriched gene sets, *G*_*i*_ and *G*_*j*_, we computed the Jaccard index (*J*) using the formula below:

1 $$ J\left({P}_i,{P}_j\right)=\frac{\left|{G}_i\cap {G}_j\right|}{\left|{G}_i\cup {G}_j\right|} $$

This evaluates to the number of genes common in the two pathways divided by the total number of genes in both pathways without repeats. Hence, Jaccard index takes values between 0 and 1, and, using this metric, the proportional similarity between two pathways can be deduced. Here, we defined two pathways to be either in crosstalk or similar based on their Jaccard scores. We relied on a cut-off of 0.60 and 0.25 to infer pathway redundancy and pathway crosstalk respectively. Since we used multiple pathway databases (KEGG [35], GO-BP [38], WikiPathways [36] and Reactome [37] pathway definitions) in our analysis, which increased the possibility of pathway redundancies, this approach allowed us to prioritize a family representative for redundant pathways, effectively eliminating sub-pathways originating from the same pathway database. To graphically illustrate the outcome of the Jaccard analysis and visually inspect the pathways for prioritization, we used the igraph R package [41] to construct pathway-pathway interaction networks as we describe later. The pathway definitions were used as the network nodes while a cut-off of 0.25 was used to insert an edge between any pathways with at least 25% common genes. Furthermore, we used greedy optimization algorithm in igraph to define clusters in a pathway-pathway interaction network.

### Inference of targeted oncogenic signalling pathways 

Using the pathway-pathway interaction networks, we applied a two-tier approach to infer biological significance. First, we relied on the 10 canonical oncogenic signalling pathways from the comprehensive pathway analysis by the TCGA Pan-Cancer Consortia [9], which are cell cycle, Hippo, Myc, Notch, NRF2, PI-3-Kinase/Akt, RTK-RaS-MAPK, TGF-β p53 and Wnt/ β-catenin signalling pathways. Among the terms identified in our enrichment analysis, we selected the terms that were semantically related to the aforementioned canonical pathways as drug-targeted signalling pathways. Subsequently, we grouped such terms into three broad clusters depicting the main cancer pathophysiologic processes: (i) cell cycle, proliferation and apoptosis, (ii) cell metastasis and invasion, and (iii) angiogenesis [42].

## Results

### Construction of drug responsive protein interaction subnetworks from transcriptome data

Breast cancer is molecularly classified into three main subtypes: luminal (A and B), triple negative and human epidermal receptor 2 positive (HER2+); based on hormone receptor (oestrogen receptor (ER) and progesterone receptor (PR) hormones) and HER2 protein expression [43]. While the datasets used in this study included representative cell lines from the three subtypes, they differ on the transcriptomic platforms used to collect the data and the drug applied. Nevertheless, we use these datasets to demonstrate that the approach proposed here captures the systemic drug effects and would be appropriate for the investigation of the pleiotropic nature of plant derived compounds. We summarise these datasets in Supplementary Table 1. In general, our datasets include luminal A (T47D, MCF-7, ZR751), triple negative (MDA-MB-231, MDA-MB-157 and MDA-MB-436) and HER2+ (MDA-MB-453) breast cancer cell lines treated with at least one of indole-3-carbinol, Withaferin A, CKI and Actein. As no luminal subtype B was covered by this study, all subtype A are referred to as ER+ throughout this discussion. The Principal Component Analysis results showing separate grouping of treatment and control samples is available as Supplementary Fig. 1. To identify affected genes, we performed differential gene expression analysis. We relied on fold change and FDR scores as cut-offs for significance, which were eventually used for data binarization for KPM analysis, as described in the Methods section. The number of differentially expressed genes for each dataset is given in Supplementary Table 2.

Network mapping and subnetwork scoring approaches have been extensively used in integrative biology field to discover active disease- and drug-specific modules in various experiments [20, 28, 44, 45]. To elucidate the molecular effects of plant derived compounds in breast cancer, we constructed the active subnetworks from transcriptome data using KeyPathwayMiner [44]. Concurrently, using the same approach and parameters, we also constructed active subnetworks from the up- and down-regulated genes separately. The number of proteins and their interactions for all the subnetwork solutions are reported in Table 1.

**Table 1:** Summary of the topological structure of subnetwork solutions indicating the number of proteins and their interactions in each dataset studied. The right part gives the subnetwork characteristics when separate subnetworks were constructed for up- and down-regulated genes

Compounds	Cell Lines	Genes	Interactions		Genes	Interactions
Actein	MDA-MB-453	829	3858	Up	327	687
				Down	455	2166
CKI	MCF-7	1332	9331	Up	933	2838
				Down	304	1676
I3C	MCF-7	1974	10,684	Up	453	1162
				Down	1399	6816
	T47D	1681	7050	Up	620	1324
				Down	959	3254
	ZR751	1403	5457	Up	545	1105
				Down	961	6323
	MDA-MB-231	93	126	Up	17	17
				Down	86	111
	MDA-MB-157	86	110	Up	18	19
				Down	75	106
	MDA-MB-436	541	1275	Up	98	120
				Down	402	932
WA	MCF-7	333	941	Up	117	353
				Down	202	564
	MDA-MB-231	998	3277	Up	456	1011
				Down	480	1208

*CKI* Compound kushen injection, *I3C* Indole-3-carbinol and *WA* Withaferin A

Overall, we observed a compound- and breast cancer subtype-specific pattern based on the number of proteins and their interactions. Thus, it is deducible from these results that the different compounds studied had substantial differential and specific effects on the activity of the underlying protein interaction networks in the disease subtypes. With the differences in the number of targeted proteins, this deduction reinforces the dominant idea that no two drugs have a similar mechanism of action in complex diseases [2, 46]. As expected, the role of molecular heterogeneity of the different breast cancer subtypes in drug response can be explicitly delineated from the sizes of the subnetworks. For instance, under indole-3-carbinol, in terms of the number of enriched genes, a relatively higher number was targeted by ER+ than TN, while the reverse was observed under Withaferin A treatment of ER+ and TN cell types (Table 1). The current drug research regime focuses on specific targeted therapy (famously defined as ‘magic bullets’) [2, 46]. However, with the increasing acceptance of the poly-pharmacologic paradigm as an effective approach in the treatment of complex diseases, our network analysis results indicate that the analysed compounds target multiple proteins simultaneously to exert their effects in a network-centric multi-targeting mechanism. This observation would be beneficial under disease conditions, particularly if the cohort of targeted proteins can be linked to or are known disease drivers.

### The drug-specific subnetworks capture key breast cancer carcinogenesis-related genes as revealed by prospective prognostic prediction using network topology analysis

An overarching question is whether the genes enriched in the subnetwork solutions have any significance in breast cancer prognosis. In therapeutic terms, effective anti-carcinogenic drug candidates are known to regulate a niche of known proto-oncogenes in a disease network. To address this, network centrality measures can be used to identify topologically important target nodes (genes) in the subnetwork solutions [47]. In disease networks under compound perturbations, such genes are significantly enriched as a result of the condition (treatment) change. In this study, with the aim to prospectively validate the constructed subnetworks, we used CytoNCA [30] to extract the top five genes based on both high betweenness and degree centralities from each subnetwork. The result from this analysis is reported in Table 2.

**Table 2:** Top 5 genes from the subnetworks of each dataset based on their betweenness and degree centrality scores, depicting compound-specific signature genes in each cell line

ACT (MDA-MB-453)	CKI (MCF-7)	I3C (MCF-7)	I3C (MDA-MB-157)	I3C (MDA-MB-231)	I3C (MDA-MB-436)	I3C (T47D)	I3C (ZR751)	WA (MCF-7)	WA (MDA-MB-231)
APP	ELAVL1	TRIM25	HNRNPL	HNRNPL	HNRNPL	HNRNPL	HNRNPL	APP	TRIM25
TRIM25	HNRNPL	ELAVL1	ESR2	ELAVL1	TRIM25	TRIM25	TRIM25	TRIM25	ELAVL1
ELAVL1	APP	ESR2	TRIM25	ESR2	ESR2	ELAVL1	ELAVL1	ESR2	APP
ESR2	TRIM25	HNRNPL	CUL3	CUL3	ELAVL1	ESR2	APP	ELAVL1	RNF4
HNRNPL	RNF4	APP	BAG3	CDH1	APP	APP	RNF4	HNRNPL	NXF1

The genes are labelled using their respective universal identifiers. *ACT* Actein, *CKI* Compound kushen injection, *I3C* Indole-3-carbinol, and *WA* Withaferin A.

Betweenness and degree centrality scores for all genes in the subnetworks are given in Supplementary Table 3. Subsequently, we analysed the top-five genes by using the KM-Express [32] tool for their association with overall survival and disease stages (median expression in normal, tumour and metastasis states).

**Fig. 2:**
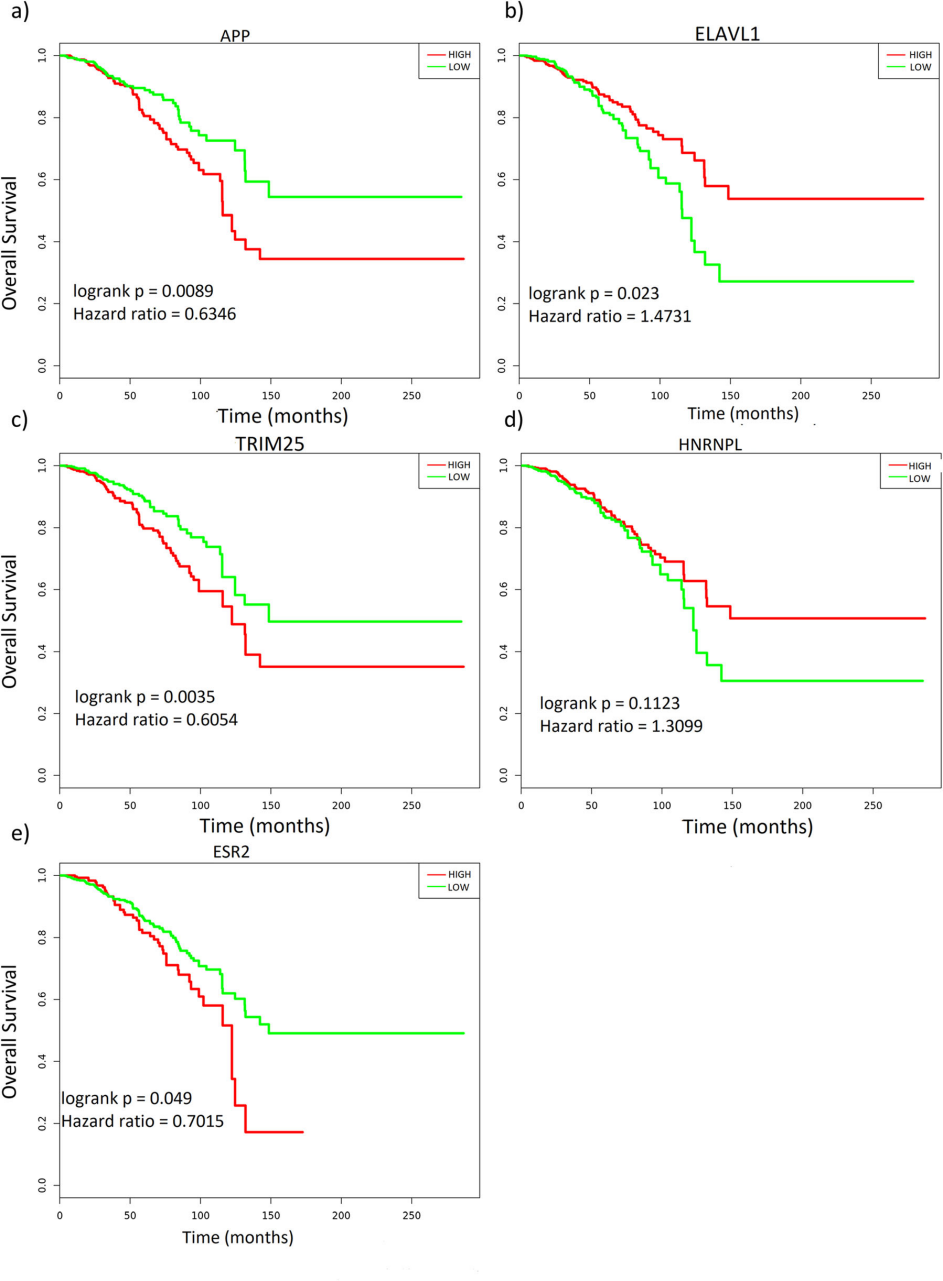
The most frequent central genes in the compound-targeted subnetworks show associations with well-defined breast cancer disease endpoints. a-e) Overall survival plots showing bifurcate (APP, ELAVL1 and TRIM25), 75% vs 25% (HNRNPL) and 75% (ESR2) gene expression in relation to patient overall survival across TCGA breast cancer datasets. ‘High’ and ‘Low’ denotes patient cohorts with high median gene expression over the follow-up period. Logrank (p-value) <0.05 was considered significant

In general, we found 11 unique genes from all the subnetworks. Five of these genes (APP, TRIM25, ELAVL1, HNRNPL and ESR2) were found to be the most frequent across all subnetworks (Table 2). Since we had allowed the parameter *K* = 5 in KPM-based subnetwork construction step, the top five genes mainly consisted of genes with non-significant differential expression but with the highest degree and betweenness centrality scores. Survival analysis found APP, TRIM25 and ELAVL1 to have significant associations with overall survival (log-rank *p*-value <0.05) in breast cancer. Overexpression of APP and TRIM25 in cancer patients was associated with low overall survival and the reverse was true for ELAVL1 (Fig. 2 a-c). In the literature, APP is a well-established cancer biomarker, a target of ADAM10, and has been strongly linked with breast cancer growth, metastasis and migration [48]. A comprehensive study identified TRIM25 as a key gene in regulating TN breast cancer metastasis [49]. ELAVL1 codes for an RNA binding protein controlling multiple facets of carcinogenesis, and literature reports show its over-expression to be associated with adverse-event free tumours [50]. Indeed, our current finding concurs that its low expression in cancer patients correlates with low overall survival and that over-expression may increase the patient overall survival. On the other hand, HNRNPL and ESR2, which have been reported to be associated with breast cancer elsewhere [51], were not significantly associated with patient survival at the median gene expression cut-off. However, further interrogation revealed their significant association with overall survival at 75% vs 25% (high vs low) and 75% gene expression cut-offs respectively (Fig. 2 d-e). From Supplementary Fig. 2 a-e, high expression levels of TRIM25 is associated with metastatic tumours while that of ELAVL1 is associated with primary tumours. The expression of APP, on the other hand, decreases in both primary and metastatic tumours., We found TRIM25 to be indirectly targeted by all the compounds, except in MDA-MB-231 under indole-3-carbinol (Fig. 2). Also, under indole-3-carbinol treatment, APP was not present amongst the top-five genes in MDA-MB-231 and MDA-MB-157, indicating a transcriptome deviation from the other TN-specific cell line, MDA-MB-436.

These findings suggest that these plant-derived compounds target gene subnetworks driven by well-established oncogenes. Importantly, the plant-based compounds exert their effects not directly through the central oncogenes but by perturbing a high number of their first neighbours. The observed protein interaction network-disease-prognosis consistency suggests that the applied method is able to capture biologically relevant protein networks and shows the potentials of the compounds used in this study to target disease-relevant networks in cancer, ostensibly permitting the constructed subnetworks for use in hypothesis generation for a compound’s mechanism of action.

### Actein, indole-3-carbinol, CKI and Withaferin A target multiple oncogenic signalling pathways which coordinate to influence cellular processes

In this section, we aimed to comprehensively catalogue drug targeted oncogenic signalling pathways and their corresponding oncogenesis processes. In summary, the following steps were followed: (i) pathway enrichment was applied to all the genes in a subnetwork, (ii) only oncogenic signalling pathways were retained, (iii) to identify and filter out redundant pathways coming from different databases, pathway-pathway correlation networks were constructed (iv) the final list of pathways was mapped on three major oncology related processes based on their semantic similarity to the 10 canonical oncogenic signalling pathways [9] (see Methods section).

**Fig. 3:**
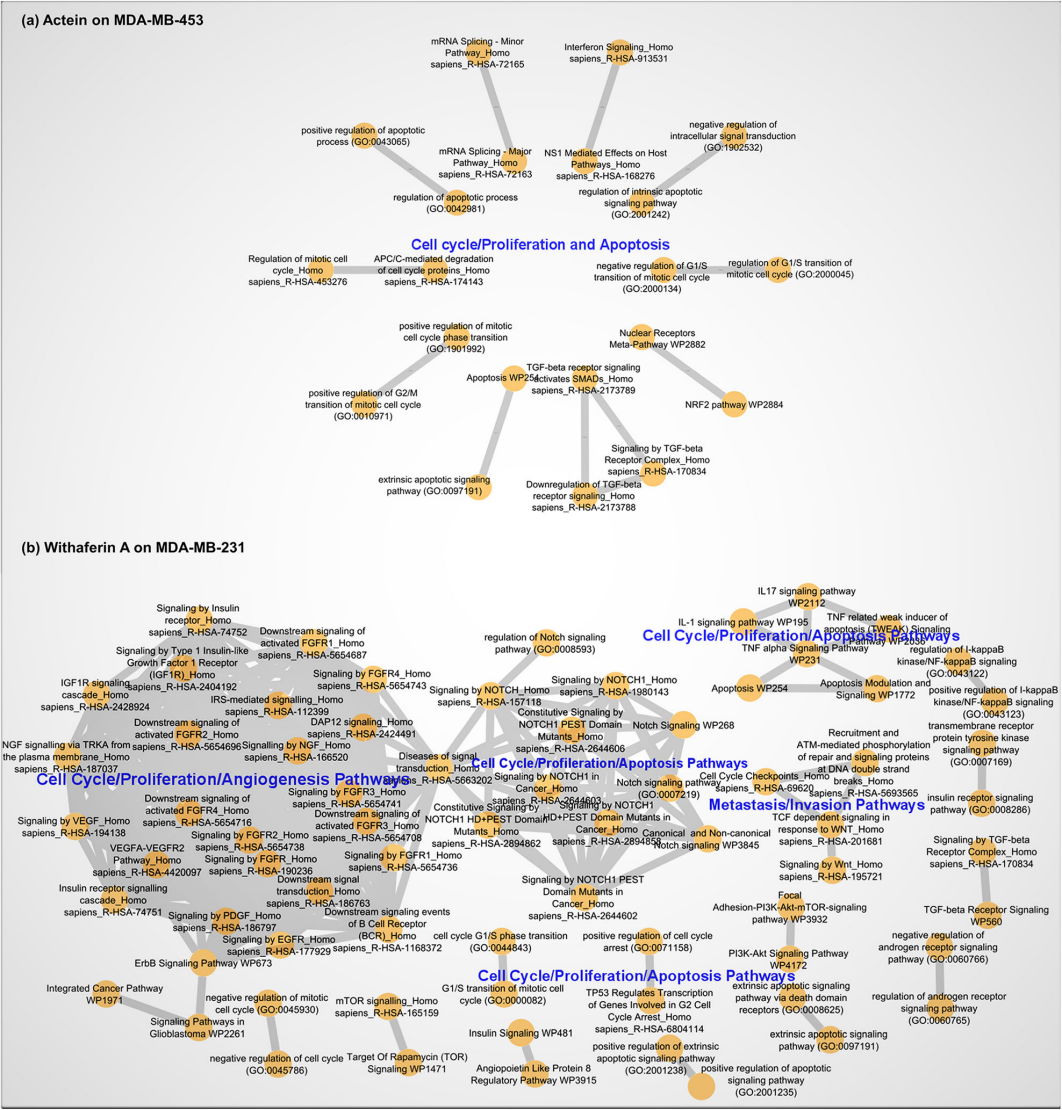
Pathway-pathway interaction networks under Actein (MDA-MB-453 cell line) and Withaferin A (MDA-MB-231 cell line) treatments. The network nodes represent individual pathways. Pathway-pathway crosstalk (Jaccard Index) ≥0.25

As described in the methods section, we performed pathway enrichment analysis using the genes in each identified subnetwork. An important factor in this systemic approach is the interconnectivity of the proteins used in pathway enrichment analysis. Thus, it is obvious that the enriched pathways are connected due to the shared targeted-network proteins. To illustrate this, first we eliminated all those pathways which were unrelated to cancer. Supplementary Table 4 and Supplementary Table 5 report the enriched pathways from this analysis. Then we constructed unweighted pathway-pathway interaction networks based on common proteins shared between different pathways. We relied on a Jaccard similarity index of at least 25% to denote pathway crosstalk (through intersecting genes) and represented this by placing an edge between them in the network. Figure 3 a-b and Supplementary Fig. 3 a-g show the networks of various drugtargeted pathways from the four drugs studied. This clustering allowed us to (i) prioritise meaningful signalling pathway terms for mapping on oncogenesis processes thus reducing redundancy (the pathways with J > 0.60), and (ii) illustrate pathway-pathway crosstalk (interdependence) in a drug-targeted network. We reckon that this approach is much simpler and precise compared to Chen et al. [52]’s gene overlap index approach for pathway prioritisation.

We observed a characteristic clustering of related pathway terms across the various enrichment results. For instance, in the actein treated MDA-MB-453 dataset, we identified 10 pathway clusters out of 21 enriched pathways; only 5 of these (NRF2, Cell cycle, Apoptosis, Interferon signalling and TGF-beta) were identified as members of the defined oncogenic signalling pathways (see Methods). An examination of the various pathway clusters from all the datasets revealed two important features: (i) the clustered pathways were either semantically related or from the same database with similar functions, as in the case of ‘NRF2’ and ‘Nuclear receptor meta-pathway’ pathways in Fig. 3 a (J > 0.60, pathway redundancy)**,** and (ii) the interacting pathways are well-known to interact in literature acting as sub-pathways through the activation of the main pathway, as in the case of ‘apoptosis’, ‘TNF’ and ‘IL17’ in Fig. 3 b (pathway crosstalk), which is expected [53]. The pathway-pathway interaction networks from the other datasets are reported in Supplementary Fig. 3 a-g.

Next, to infer biological significance, we applied a two-tier approach. First, we relied on the predefined canonical oncogenic signalling pathways (see Methods section) [9] for the concise terms. Additionally, though not captured in the TCGA [9] analysis of the most frequently mutated canonical oncogenic signalling pathways since it is a response mechanism to foreign system, the role of the immune system signalling as a secondary response mechanism in cancer is significant and can be attributed to the inhibition/promotion of tumour initiation and metastasis in advanced cases. Thus, immune system related pathway terms were also included in the analysis results based on the known physiological roles of both the pathways and their enriched genes. Subsequently, we used pathway enrichment analysis results from the up−/down-regulated subnetworks (Supplementary Table 5) to assign these pathways as either up- or down-regulated. Eventually, with clear pathway clusters and only canonical-signalling-pathways relevant non-redundant terms, we mapped the resulting pathway terms on the three categories derived from major oncogenesis processes: (i) cell cycle, proliferation and apoptosis, (ii) cell metastasis and invasion, and (iii) angiogenesis. However, given the overlapping roles different pathways perform in biological systems, deciphering the affected processes is not straightforward. Therefore, to assign a pathway to either of the three groups, we looked up for the functional role(s) of the associated genes (both up- and down- regulated) in UniProtKB [54] database. To deduce the targeted biological processes, we relied on those genes whose molecular functions match the biological roles of the pathways provided in literature. Table 3 details the results of this grouping. To illustrate this approach, we provide a detailed description of the grouping as applied to the actein treated MDA-MB-453 cell line in Supplementary Table 6 using enrichment results from Supplementary Table 5 and the pathway-pathway interaction networks (Fig. 3 a, b and Supplementary Fig. 3a-g).

**Table 3:** Mapping of targeted signaling pathways on canonical oncogenic pathways based on related cancer pathophysiologic processes

Drug	Carcinogenesis process				
	Cell Line	Activity	Cell cycle/Proliferation and Apoptosis	Metastasis and invasion	Angiogenesis
ACT	MDA-MB- 453	Down	Intrinsic Pathway for Apoptosis PTK6 Regulates Cell Cycle Interferon Signaling	–	–
		Up	PI3K-Akt-mTOR NRF2 pathway TGF-beta Signaling Pathway	–	–
CKI	MCF-7	Down	p53 signaling pathway regulation of intrinsic apoptotic signaling pathway	–	–
		Up	PI3K-AKT-mTOR signaling pathway and therapeutic opportunities EGF/EGFR Signaling Pathway NRF2 pathway Fc epsilon RI signaling pathway T cell receptor signaling pathway B cell receptor signaling pathway	Canonical and Non-Canonical TGF-B signaling	VEGFA-VEGFR2 Signaling Pathway
WA	MCF-7	Down	p53 signaling pathway NF-kB activation through FADD/RIP-1 pathway mediated by caspase-8 and − 10 Interferon Signaling Cytokine Signaling in Immune system	–	TGF-beta Signaling Pathway
		Up	NRF2 pathway MAPK Signaling Pathway p53 signaling pathway intrinsic apoptotic signaling pathway	–	–
	MDA-MB-231	Down	NRF2 pathway MAPK signaling pathway ErbB Signaling Pathway p53 signaling pathway TGF-beta Signaling Pathway Notch Signaling Pathway IL-4 Signaling Pathway IL17 signaling pathway	TCF dependent signaling in response to WNT	–
		Up	PI3K-Akt Signaling Pathway Interferon Signaling TNF signaling pathway	Inflammatory Response Pathway	VEGFA-VEGFR2 Signaling Pathway Notch (U) TGF-beta Signaling Pathway
I3C	MCF-7	Down	TP53 Regulates Transcription of Cell Cycle Genes Signaling by EGFR Apoptosis PI3K-AKT-mTOR signaling pathway and therapeutic opportunities MAPK Signaling Pathway Wnt Signaling Pathway and Pluripotency T-Cell Receptor and Co-stimulatory Signaling TNF alpha Signaling Pathway	TGF-beta Receptor Signaling	–
		Up	Apoptosis regulation of cell cycle	–	–
	T47D	Down	Cell Cycle, Mitotic ErbB Signaling Pathway PI3K-Akt Signaling Pathway Chemokine signaling pathway	Signaling by NOTCH1 in Cancer Wnt Signaling Pathway and Pluripotency TGF-beta Signaling Pathway	VEGFA-VEGFR2 Signaling Pathway PDGF Pathway
		Up	RIG-I-like Receptor Signaling Apoptosis MAPK Signaling Pathway Interferon gamma signaling TGF-beta Signaling Pathway	–	–
	ZR751	Down	EGF/EGFR Signaling Pathway Notch Signaling Pathway TGF-beta Signaling Pathway regulation of apoptotic process Negative regulators of RIG-I/MDA5 signaling	Wnt Signaling Pathway and Pluripotency	VEGFA-VEGFR2 Signaling Pathway
		Up	Interferon Signaling NRF2 pathway Apoptosis MAPK Signaling Pathway	–	–
	MDA-MB-231	Down	–	Pathways Regulating Hippo Signaling	VEGFA-VEGFR2 Signaling Pathway
		Up	NRF2 pathway	–	–
	MDA-MB-436	Down	ErbB Signaling Pathway PI3K-Akt Signaling Pathway MAPK Signaling Pathway	Wnt Signaling Pathway and Pluripotency Hippo(D) T-Cell Receptor and Co-stimulatory Signaling	PDGF(D) TGF-beta Signaling Pathway
		Up	Apoptosis-related network due to altered Notch3 in ovarian cancer TGF-beta Signaling Pathway Activated TLR4 signalling	–	–

Three major oncological processes defining the diverse molecular processes associated with carcinogenesis were used to deduce biological roles of the various enriched oncological signaling pathways

## Discussion

Systems pharmacology has evolved as a data-driven approach to bridge the gap between the increasing amounts of compound/drug perturbation data and drug discovery through systematic evaluations [46, 55]. It gives new perspectives to drug/compound treated clinical and experimental publicly available omics data through well-grounded bioinformatics data analysis pipelines, speeding up the rate of understanding of the molecular mechanisms of action to identify targets of drug candidates [1, 2, 56]. In this study, we developed and implemented a computational analysis framework that relies on mapping transcriptome data on protein interactome and constructing targeted subnetworks, and subsequent mapping of enriched pathways in the subnetworks on carcinogenesis processes (Fig. 1). For poly-pharmacologic compounds, this approach projects the cellular behaviour in response to treatment on a physical interaction network; thereby, simplifying inference of mechanism of action from omics data. While it would have been important to include more studies to augment the results obtained by this approach, most of the available transcriptome datasets available did not pass the quality control step. Finally, we showed that the findings from our approach augments initial studies on the compounds and propose new processes that were not reported in those studies. Below, we discuss the main findings with literature evidences on the studied compounds.

Actein has been widely studied in breast cancer due to its effects on various biological processes in various cancers [11, 57–59]. Initial findings by Einbond et al. (2007) [11] using the same dataset demonstrated a dosage dependent activation of integrated stress response pathway, cell survival and apoptosis pathways as the main mechanisms targeted by the compound. In this study, cell death and cell cycle roles of TGF-β, PI3K-Akt-mTOR and NRF2 pathways were up-regulated while proliferation roles of TGF-β pathway were down-regulated. Additionally, tumour microenvironment regulation through interferon signalling pathway was down-regulated (Table 3). Available reports on breast and other cancers indicate that actein targets cell apoptosis [58, 60], cell adhesion [59] and migration [59, 60]. This analysis showed actein to target oncogenic signalling pathways mainly regulating cell cycle, proliferation and apoptosis processes in this cell type, which clearly captures the initial findings [11] and proposes more mechanisms that were not captured by the study.

CKI is an ancient formulation in the Chinese pharmacopoeia, and mixed results have been reported in breast cancer [61]. The group by Qu et al. (2016) [13] showed that cell cycle and other cell growth related pathways are the main potential targets of CKI. Here, we found CKI to down-regulate p53 pathway, which is in line with a previous observation of p53 independent apoptotic cell death [13], and up-regulate RTK-RaS-MAPK (EGFR, p38 and ErbB), PI3K-Akt-mTOR, NRF2 and TGF-beta pathways in MCF-7. These pathways regulate cell proliferation and apoptosis (p53, RTK-RaS-MAPK, PI3K-Akt-mTOR and NRF2) and metastasis/invasion (TGF- β). Moreover, CKI also targets angiogenesis and tumour microenvironment regulating pathways through VEGFA/VEGFR2 and cytokine signalling (B cell receptor, T cell receptor and FC-epsilon signalling) respectively (Supplementary Table 5), which is consistent with a previous finding [62]. Other reports have shown that CKI directly regulates cell migration [63]; and apoptosis in breast cancer [62]. Cell cycle, proliferation and apoptosis, metastasis/invasion, and angiogenesis were proposed here as the potentially targeted carcinogenesis processes in this cell line, which agrees well with the initial findings [13] (Table 3).

The therapeutic effectiveness of indole-3-carbinol is well defined in oestrogen receptor driven cancers [64, 65]. Caruso et al. (2014) [14] showed that I3C mainly acts by targeting the pro-apoptotic aryl hydrocarbon receptor mediated by increased oxidative stress in ER+ cell lines. In ER + cell types, we mapped the pathways on cell proliferation and apoptosis (Wnt, cell cycle, Notch and TGF-β) and invasion/metastasis (TGF-β, Wnt and Notch). Characteristically, TGF-β regulating metastasis/invasion was down-regulated in T47D and MCF-7 while its cell death promoting role was up-regulated in T47D and down-regulated in ZR751 (Table 3 and Supplementary Table 5). All the three categories of carcinogenesis processes were targeted (Table 3). The role of indole-3-carbinol on TN is less studied, however low efficacy in this subtype has been noted [14]. Accordingly, here no oncogenic signalling pathway was enriched in the MDA-MB-157 subnetwork, illustrating an indole-3-carbinol -specific non-responsive subtype. This tumour subtype is known to be resistant to most chemotherapeutic interventions [66]. Nonetheless, more MDA-MB-436 signalling pathways were targeted by indole-3-carbinol than in MDA-MB-231 subtype (Supplementary Table 5); and they control carcinogenesis through cell cycle, proliferation and apoptosis, metastasis/invasion, and angiogenesis processes (Table 3). In effect, we identified more potentially targeted pathways than reported in the original work and showed that disease specific pathways are also targets of the compound in TN subtypes [14].

The characteristic anti-cancer effects of Withaferin A is well anchored in scientific reports [67–70] and specifically in breast cancer [12, 68, 71, 72]. WA was previously found to mainly remodel TN metastatic molecular phenotype to ER+ [12] non-metastatic phenotype. Here, RTK-RaS-MAPK, TGF-β, NRF2 and p53 oncogenic signalling pathways were targeted in both TN and ER+. Tumour subtype specificity on Wnt, Notch, VEGFA-VEGFR2 and PI3K-Akt-mTOR in TN and cytokines in ER+ were observed (Table 3). Moreover, cytokine mediated signalling in both cells was also targeted. The up-regulation of NRF2 pathway genes as observed is consistent with in vivo findings of induced oxidative stress in the two cell lines [68, 73]. These results illustrated multi-targeting of several carcinogenesis processes, including cell proliferation and death, metastasis/invasion and angiogenesis (Table 3) in both TN and ER+ associated with phenotypes reported in in vitro studies [12, 68, 71, 72, 74]. Thus, besides mainly targeting tumour metastasis, we show that WA could potentially target more cancer specific pathways in both ER+ and TN as shown in Table 3.

Whereas this work attempts to associate the various targeted networks with carcinogenesis processes to explain the mechanism of action of poly-pharmacologic compounds, a major limitation arises on enumerating their therapeutic values. For instance, enrichment of a pathway in either up- or down-regulated subnetworks may not necessarily be directly translated as activation or inactivation of the related pathway-defined cellular process, as the same process may be targets of other co−/dys-regulated pathways by the same drug. In vitro reports on the activity of the different compounds on cell lines [11–14] showed agreements with our findings. However, we suggest that the new processes identified by this study need further validation studies. To increase the robustness of this approach, we propose future integration of more omics data to provide a more precise picture on the exact mechanism of action of these compounds [75].

Another challenge experienced in this approach is the un-directionality of protein interactomes. Thus, given the inherent directionality in signalling pathways, our future studies will incorporate directed networks from an ensemble of databases, by drawing on their comprehensiveness to construct all-inclusive interaction networks.

Additionally, given the poly-pharmacologic properties found here, simulations on the effect of different combinations to determine synergistic and antagonistic combinations and side-effects would provide more information. Regan-Fendt et al. [76] recently developed a computational drug combination analysis using transcriptome data and disease specific root genes for malignant melanoma and successfully predicted vemurafenib and tretinoin as synergistic therapeutic combinations. Variants of this approach, for instance, modelling the active drug subnetworks using deep learning, could be applied to systematically predict drug combinations and side-effects for precision medicine applications in pre-clinical drug research for complex diseases [8, 55].

## Conclusion

Literature evidence from other in vitro studies on both breast and other cancers were shown to support some of our predictions on the systemic effects of the studied compounds. This suggests the method may be valuable in identifying the systemic molecular effects of pleiotropic compounds during drug screening. Additionally, it may be used to select the appropriate compound based on targeted pathways or biological processes associated with disease. However, more in vitro studies are needed to validate the predictions in the respective cell types before wider adoption of this methodology.

Overall, this study generated two main outputs: (i) proposed a data-driven framework for elucidating the mechanism of action of pleiotropic natural products using transcriptome data and protein interactome and (ii) demonstrated that plant-derived drugs (actein, indole-3-carbinol, withaferin A and CKI) are capable of simultaneously regulating multiple carcinogenesis processes in breast cancer. Thus, this network-centric method can extract subtle systemic drug effects on cellular pathways and provides a better approach to the abortive exquisite ‘target’ approach in studying poly-pharmacologic compounds. Although breast cancer datasets were used to prove the concept, the approach can also be easily applied to other cancers. We anticipate that the proposed framework will be instrumental in accelerating evaluation of poly-pharmacologic compounds for applications in pre-clinical drug efficacy research for oncology precision medicine and other complex diseases.

## Supplementary Information

**Additional file 1: Supplementary Fig. 1.** Principal component analysis (PCA) results of transcriptome samples for each dataset illustrating the distribution of variance in the first two components considered for sample separation. PC1: principal component 1, PC2: principal component 2. (a) actein on MDA-MB-453, (b) CKI on MCF-7, (c) Indole-3-Carbinol on MCF-7, (d) Indole-3-Carbinol on MDA-MB-231, (e) Indole-3-Carbinol on MDA-MB-436, (f) Indole-3-Carbinol on T47D, (g) Indole-3-Carbinol on ZR751, (h) Withaferin A on MCF-7 and (i) Withaferin A on MDA-MB-231.

**Additional file 2: Supplementary Fig. 2.** Average gene expresion profiles of most frequent central genes in the compound-targeted subnetworks based on TCGA datasets. a-e) Box-plots showing gene-phenotype (primary, normal and metastatic) association.

**Additional file 3: Supplementary Fig. 3.** Pathway-pathway interaction networks based on shared enriched genes illustrating functional pathway cross-talk. a-f: represents networks of pathways targeted by CKI on MCF-7, I3C on MCF-7, I3C on MDA-MB-436, I3C on T47D, I3C on ZR751 and WA on MCF-7. CKI: Compound Kushen Injection, I3C: Indole 3-Carbinol, WA: Withaferin A.

**Additional file 4: Supplementary Table 1.** Summary of the transcriptome datasets used and the molecular profiles of the cell lines. The columns Controls and Treatments list the number of samples in each case. (HER2+: human epidermal receptor 2 positive, ER+: Oestrogen receptor positive, TN: triple negative, AC: adenocarcinoma, IDC: invasive ductal carcinoma, MC: medullary carcinoma, Wt: wild type, Mut: Mutant, Del: deleted).

**Additional file 5: Supplementary Table 2.** Summary of the differential expression analysis results. The number of differentially expressed genes under the respective plant-derived drugs/compounds are given in the table. DEG: Differentially expressed genes, FDR: False discovery rate, FC: Fold change.

**Additional file 6: Supplementary Table 3.** Results of subnetwork betweenness- and degree centrality analysis.

**Additional file 7: Supplementary Table 4.** Pathways enriched in whole subnetworks. FDR < 0.05.

**Additional file 8: Supplementary Table 5.** Enriched pathways in up- and down-regulated subnetworks. FDR < 0.05.

**Additional file 9: Supplementary Table 6.** An example of Actein targeted oncogenesis processes illustrating the approach used in grouping the oncogenic signaling pathways into different cancer pathophysiological processes based on the pathways’ enriched genes.

## Data Availability

The transcriptome data are publicly available on https://www.ncbi.nlm.nih.gov/geo/ through accession numbers GSE53049 [12], GSE7848 [11], GSE78512 [13] and GSE55897 [14]; the protein-protein interaction data were derived from BioGRID [29] and the oncogenic signalling pathway data information were sourced from a recently published TCGA paper [9]. All codes are available upon request while all open source softwares used at each stage are cited in the manuscript.

## Abbreviations

CKI: Compound kushen injection
WA: Withaferin A
I3C: Indole-3-carbinol
HER2+: Human epidermal receptor 2 positive
ER+: Oestrogen receptor positive
PR +: Progesterone receptor positive
TN: Triple negative breast cancer
LA: Luminal A breast cancer
PPIN: Protein-protein interaction network
KPM: KeyPathwayMiner
TCGA: The Cancer Genome Atlas
TGF-β: Tumour growth factor- beta
NRF2: Nuclear factor erythroid 2 related factor 2
TNF: Tumour necrosis factor
IL17: Interleukin 17
PI3K: Phosphoinositide-3 kinase
mTOR: Mammalian target of rapamycin
EGFR: Epidermal growth factor receptor
RTK: Receptor tyrosine kinase
RaS: Rat sarcoma
MAPK: Mitogen activated protein kinase
Wnt: Wingless and Int-1
VEGFA: Vascular endothelial growth factor A
