# Supplementary material for: A systems pharmacology approach based on oncogenic signalling pathways to determine the mechanisms of action of natural products in breast cancer from transcriptome data
Source: BMC Complement Med Ther. 2021 Jun 30;21:181. doi: 10.1186/s12906-021-03340-z (PMC8244196; doi:10.1186/s12906-021-03340-z)
Supplement: Supplementary file 1 — Additional file 1: Supplementary Fig. 1. Principal component analysis (PCA) results of transcriptome samples for each dataset illustrating the distribution of variance in the first two components considered for sample separation. PC1: principal component 1, PC2: principal component 2. (a) actein on MDA-MB-453, (b) CKI on MCF-7, (c) Indole-3-Carbinol on MCF-7, (d) Indole-3-Carbinol on MDA-MB-231, (e) Indole-3-Carbinol on MDA-MB-436, (f) Indole-3-Carbinol on T47D, (g) Indole-3-Carbinol on ZR751, (h) Withaferin A on MCF-7 and (i) Withaferin A on MDA-MB-231. [file 12906_2021_3340_MOESM1_ESM.pdf]

**Figure S1:** Principal component analysis (PCA) results of transcriptome samples for each dataset illustrating the distribution of variance in the first two components considered for sample separation. PC1: principal component 1, PC2: principal component 2. (a) actein on MDA-MB-453, (b) CKI on MCF-7, (c) Indole-3-Carbinol on MCF-7, (d) Indole-3-Carbinol on MDA-MB-231, (e) Indole-3-Carbinol on MDA-MB-436, (f) Indole-3-Carbinol on T47D, (g) Indole-3-Carbinol on ZR751, (h) Withaferin A on MCF-7 and (i) Withaferin A on MDA-MB-231.

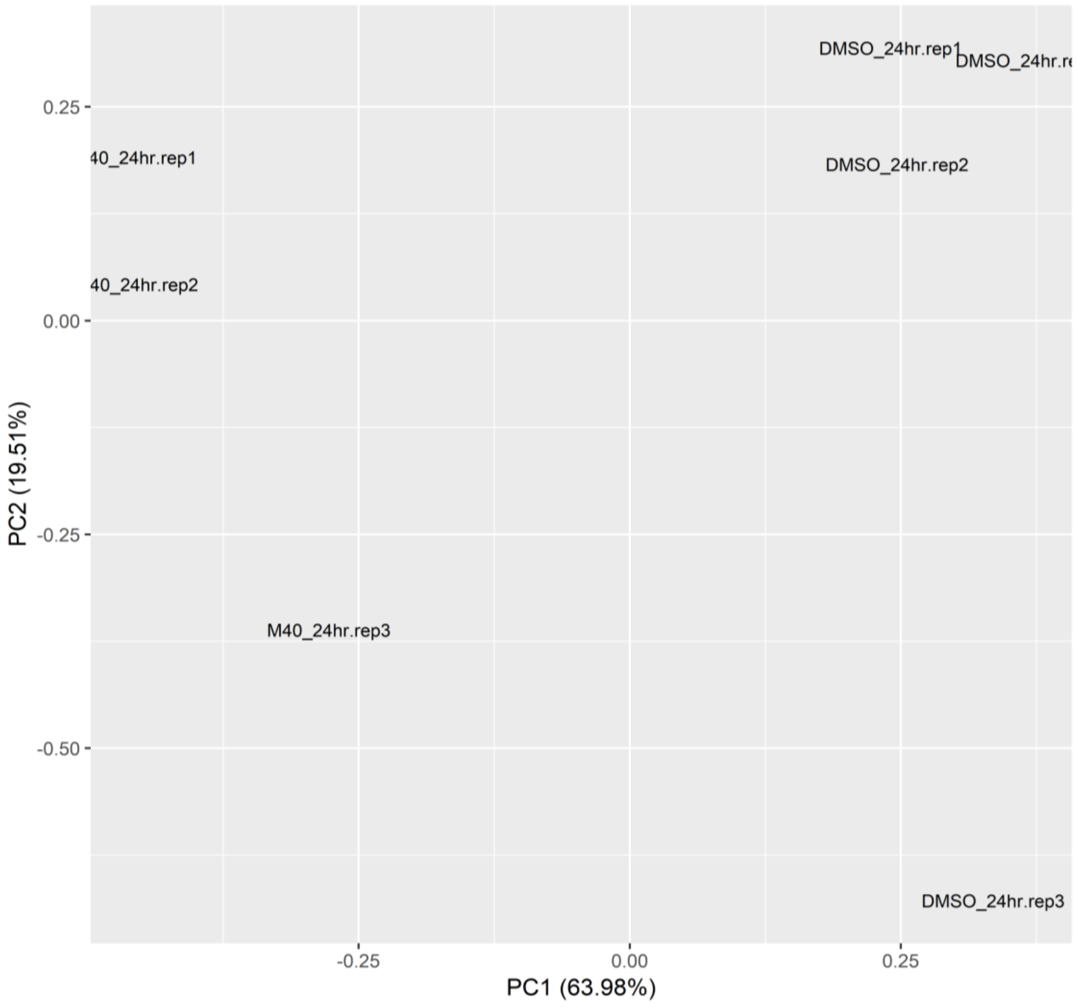


**(a)** Actein on MDA-MB-453.

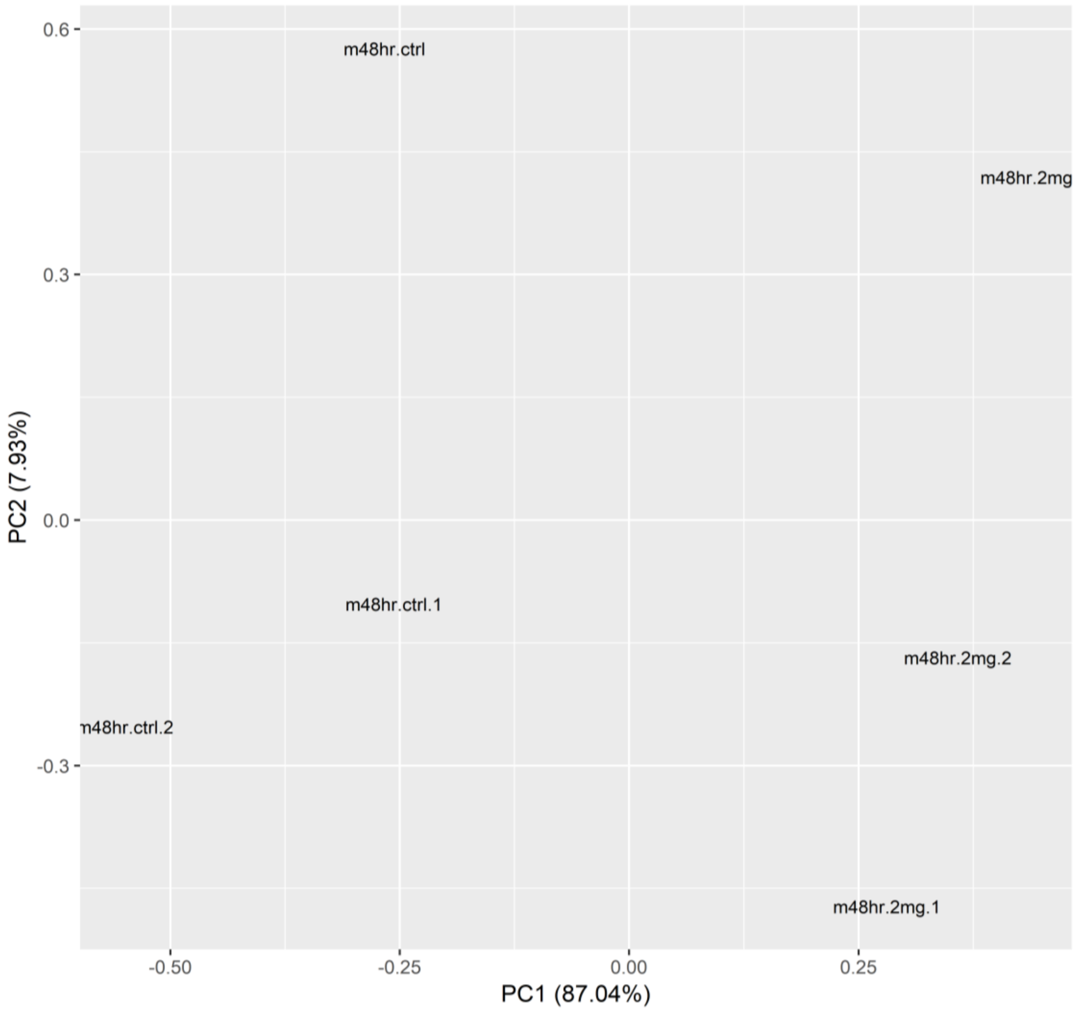

m48hr.ctrl
m48hr.2mg
m48hr.ctrl.1
m48hr.2mg.2
m48hr.ctrl.2
m48hr.2mg.1
0.6
0.3
0.0
-0.3
-0.50
-0.25
0.00
0.25
PC1 (87.04%)
PC2 (7.93%)


**(b)** CKI on MCF-7.

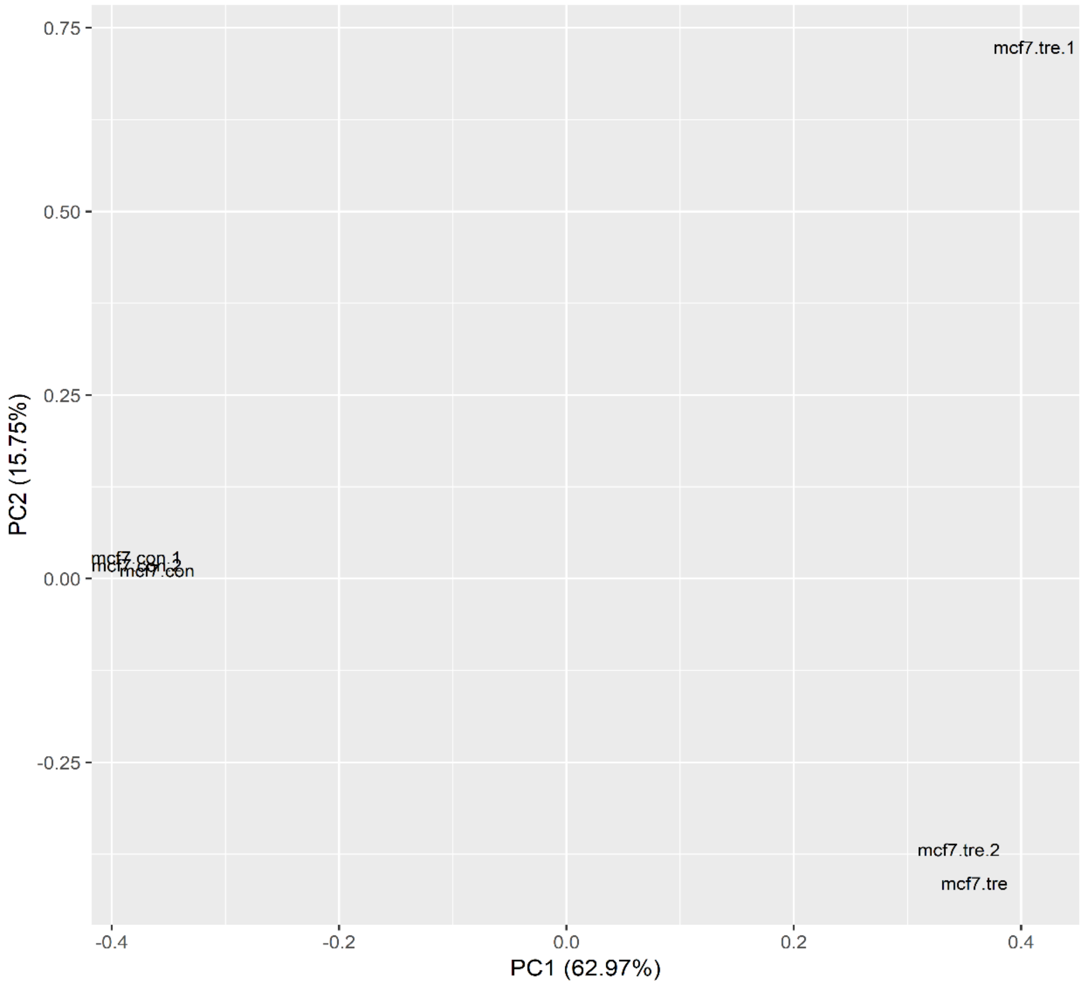


**(c)** Indole-3-Carbinol on MCF-7.

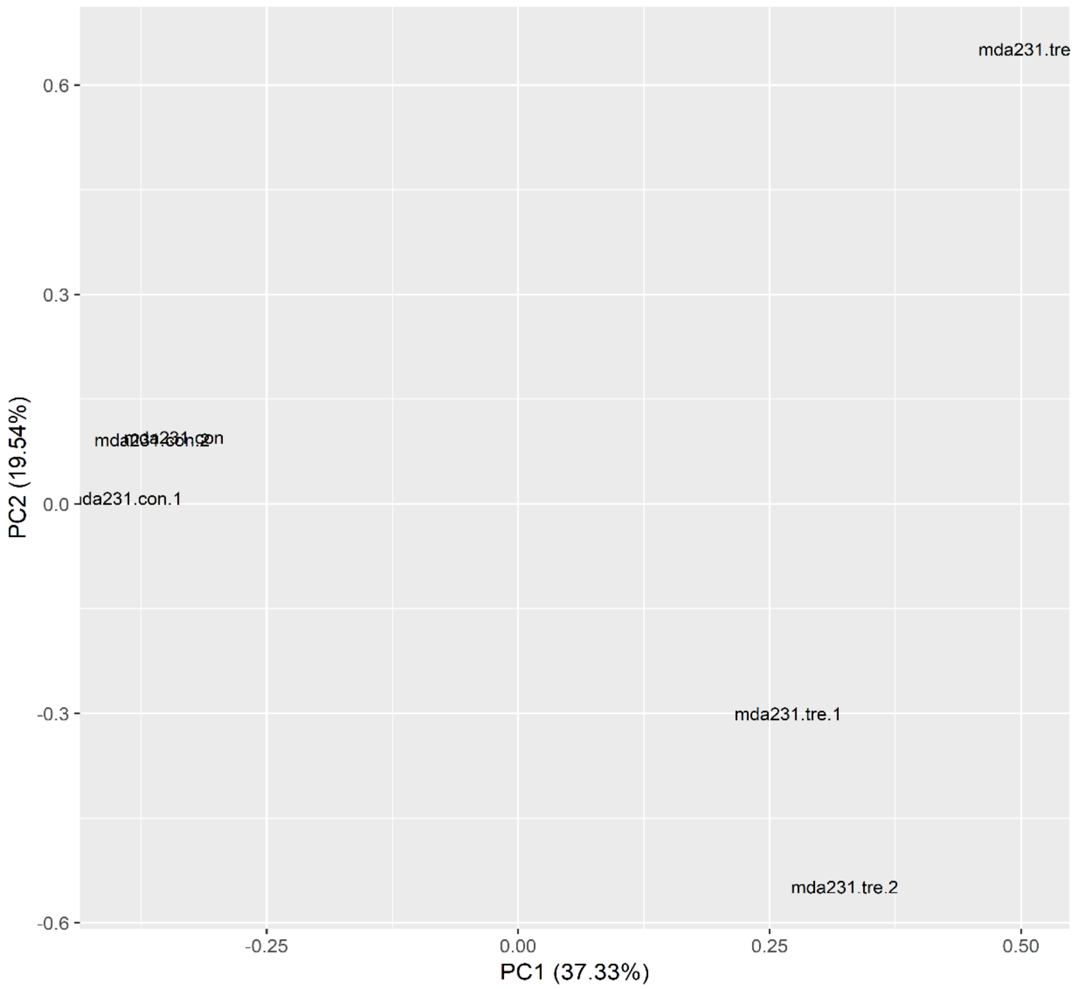


**(d)** Indole-3-Carbinol on MDA-MB-231.

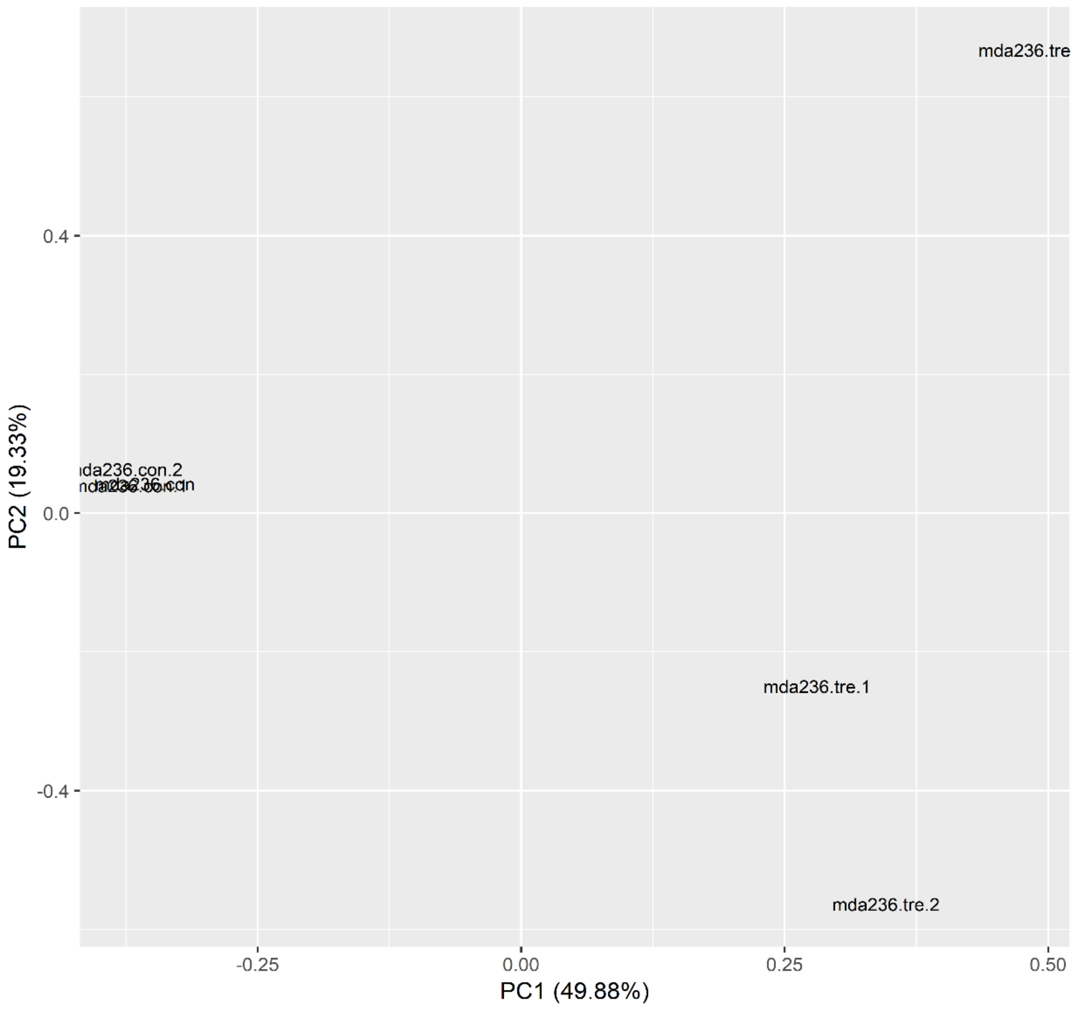


**(e)** Indole-3-Carbinol on MDA-MB-436.

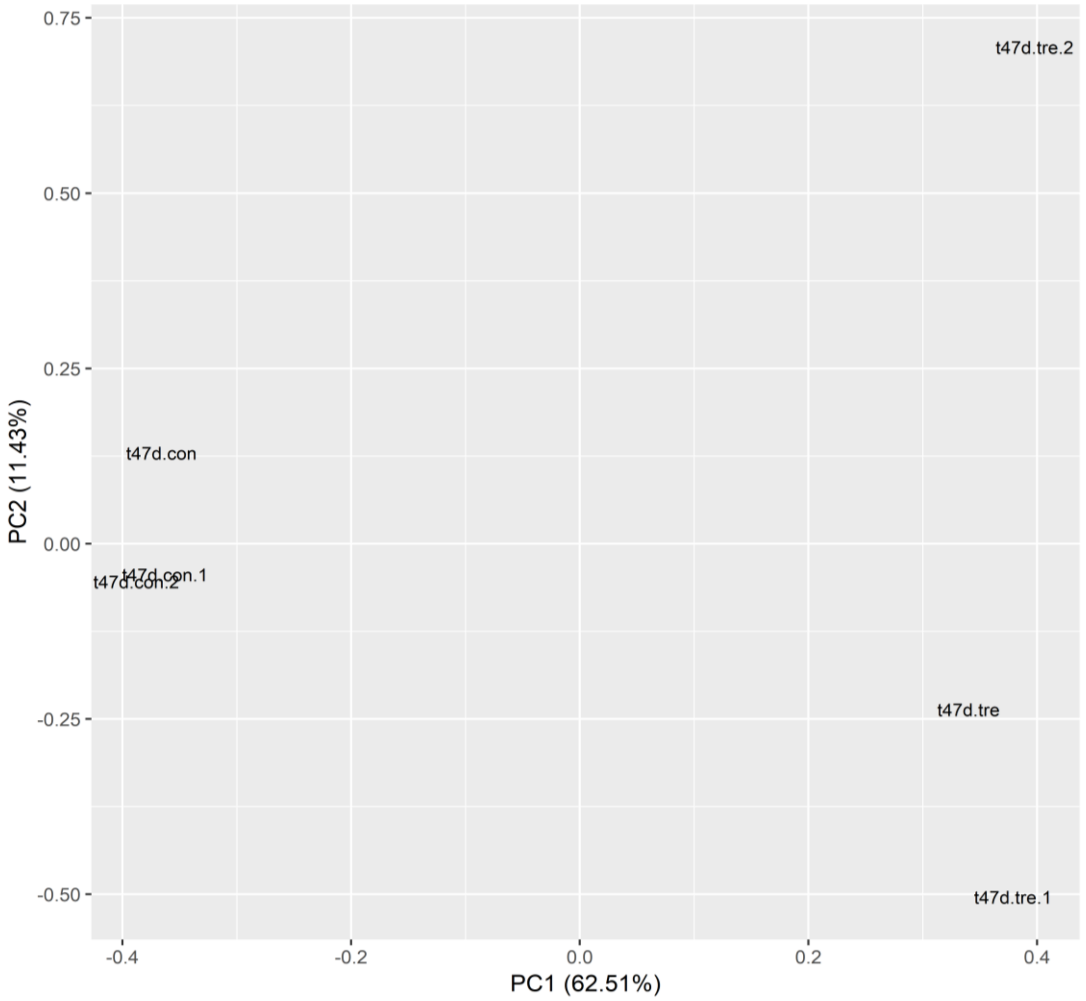


**(f)** Indole-3-Carbinol on T47D.

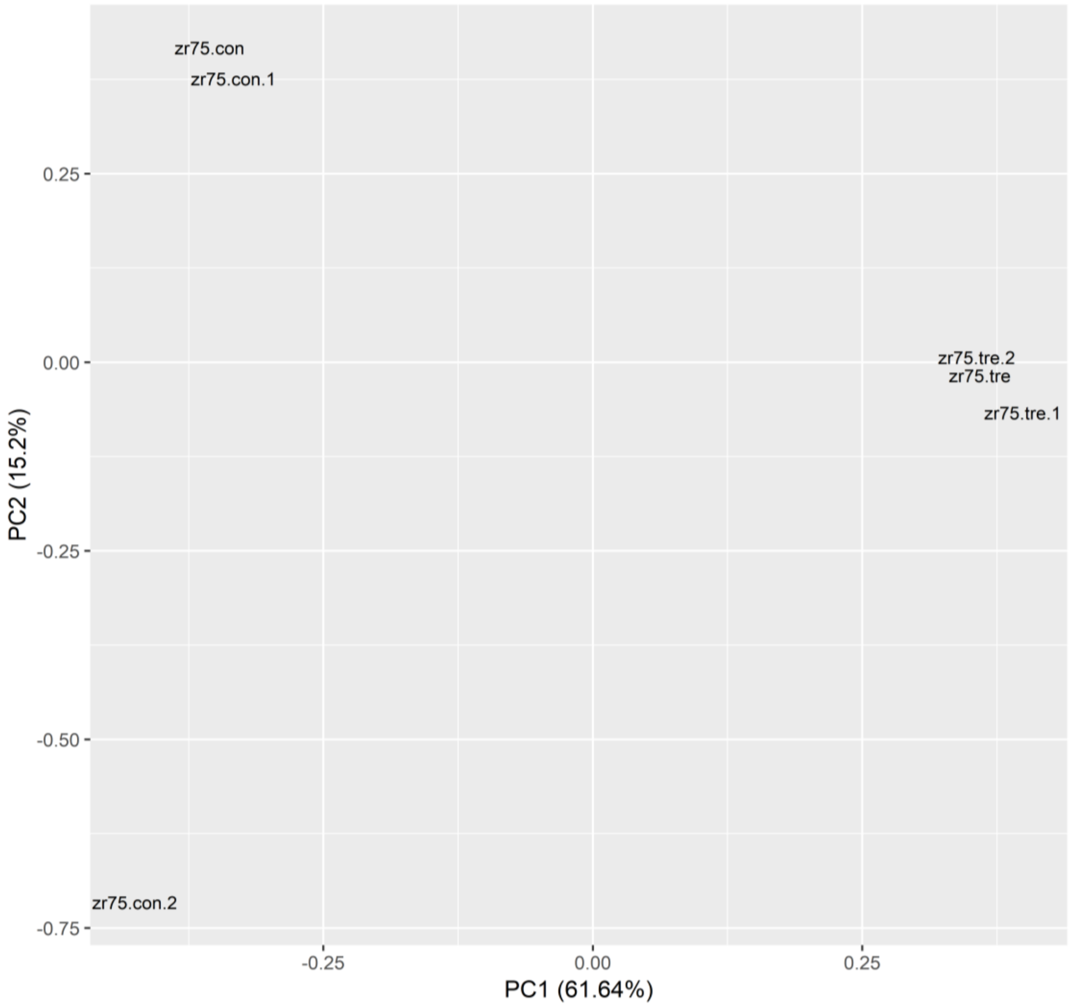


**(g)** Indole-3-Carbinol on ZR751.

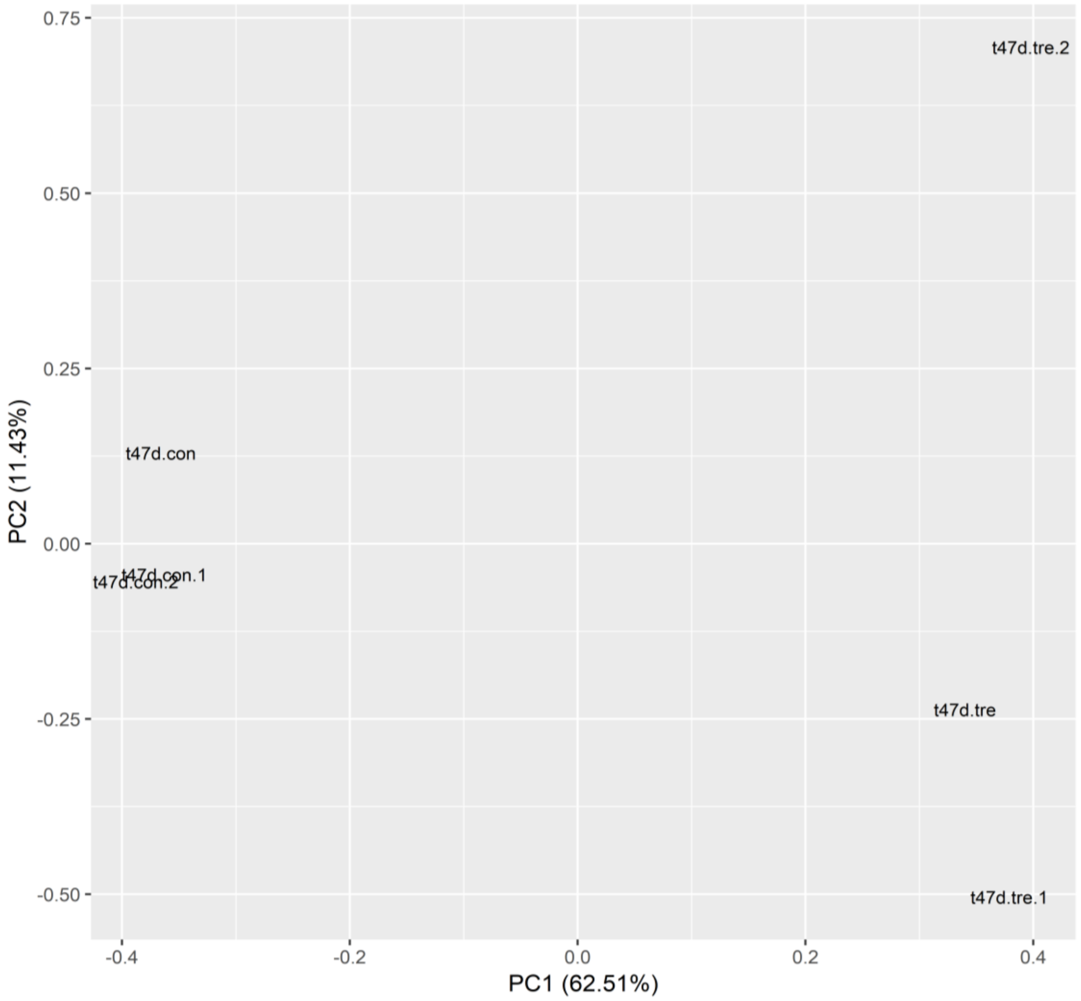


**(h)** Withaferin A on MCF-7.

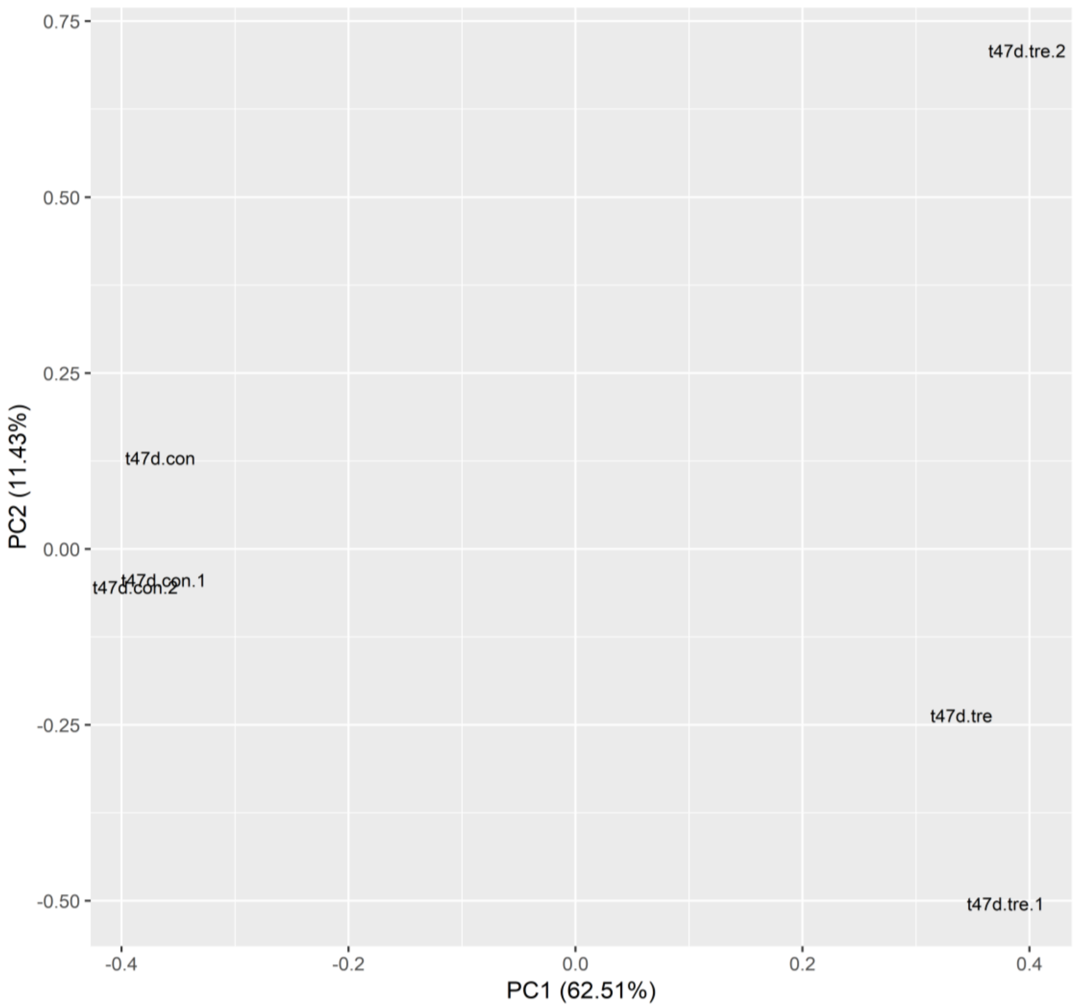


**(i)** Withaferin A on MDA-MB-231.
