# Supplementary material for: A systems pharmacology approach based on oncogenic signalling pathways to determine the mechanisms of action of natural products in breast cancer from transcriptome data
Source: BMC Complement Med Ther. 2021 Jun 30;21:181. doi: 10.1186/s12906-021-03340-z (PMC8244196; doi:10.1186/s12906-021-03340-z)
Supplement: Supplementary file 2 — Additional file 2: Supplementary Fig. 2. Average gene expresion profiles of most frequent central genes in the compound-targeted subnetworks based on TCGA datasets. a-e) Box-plots showing gene-phenotype (primary, normal and metastatic) association. [file 12906_2021_3340_MOESM2_ESM.pdf]

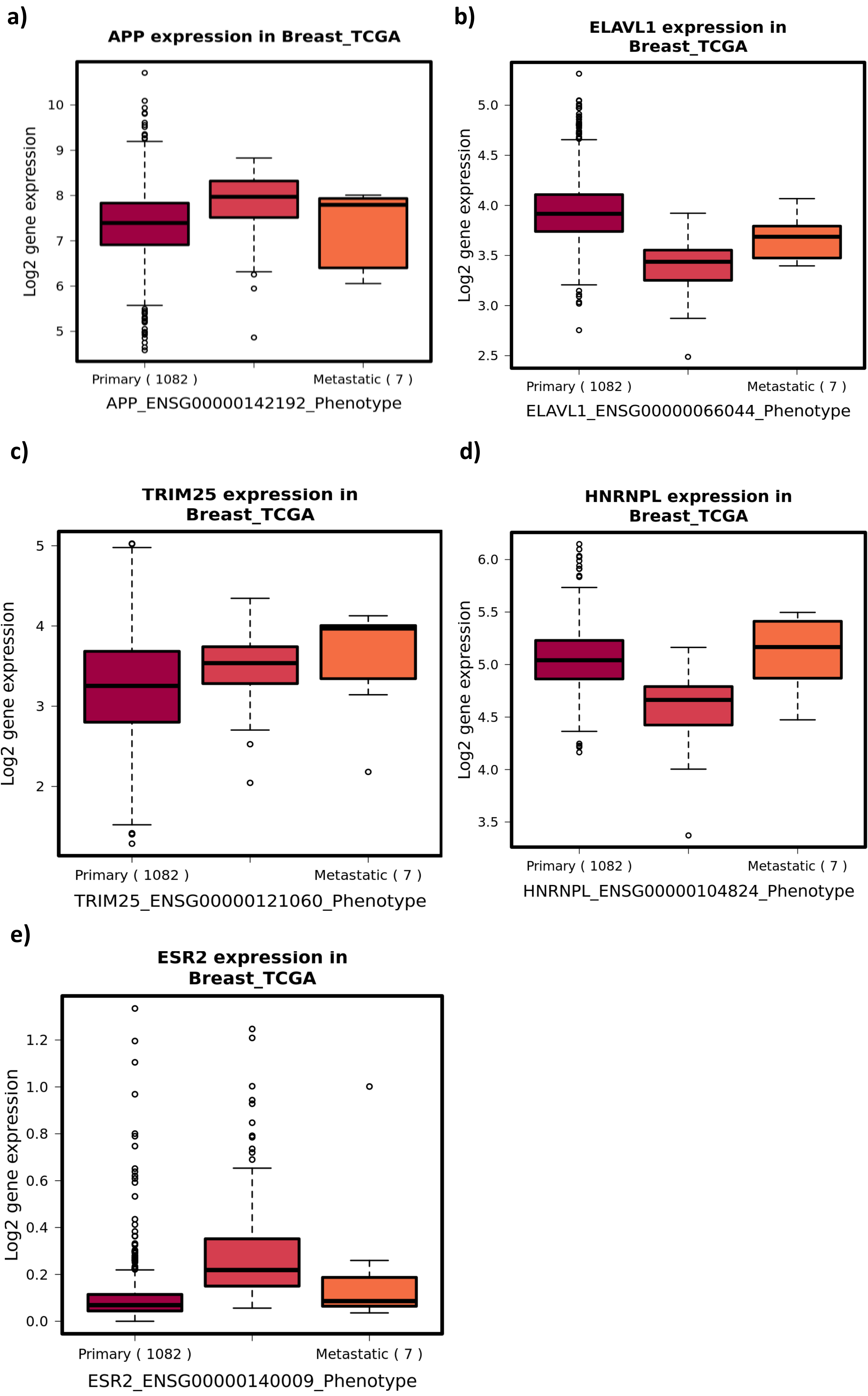
a)
APP expression in Breast_TCGA
Log2 gene expression
10
9
8
7
6
5
Primary (1082)
Metastatic (7)
APP_ENSG00000142192_Phenotype
b)
ELAVL1 expression in Breast_TCGA
Log2 gene expression
5.0
4.5
4.0
3.5
3.0
2.5
Primary (1082)
Metastatic (7)
ELAVL1_ENSG00000066044_Phenotype
c)
TRIM25 expression in Breast_TCGA
Log2 gene expression
5
4
3
2
Primary (1082)
Metastatic (7)
TRIM25_ENSG00000121060_Phenotype
d)
HNRNPL expression in Breast_TCGA
Log2 gene expression
6.0
5.5
5.0
4.5
4.0
3.5
Primary (1082)
Metastatic (7)
HNRNPL_ENSG00000104824_Phenotype
e)
ESR2 expression in Breast_TCGA
Log2 gene expression
1.2
1.0
0.8
0.6
0.4
0.2
0.0
Primary (1082)
Metastatic (7)
ESR2_ENSG00000140009_Phenotype
