# Supplementary material for: A systems pharmacology approach based on oncogenic signalling pathways to determine the mechanisms of action of natural products in breast cancer from transcriptome data
Source: BMC Complement Med Ther. 2021 Jun 30;21:181. doi: 10.1186/s12906-021-03340-z (PMC8244196; doi:10.1186/s12906-021-03340-z)

(a) Compound Kushen Injection on MCF-7

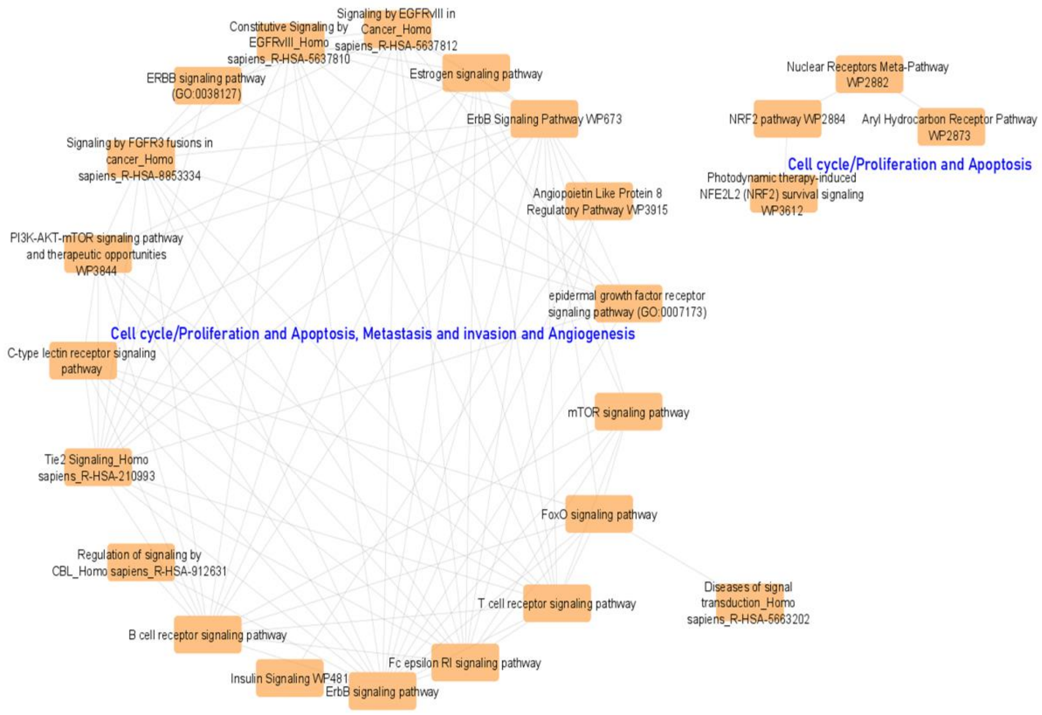

(b) Indole 3-Carbinol on MCF-7

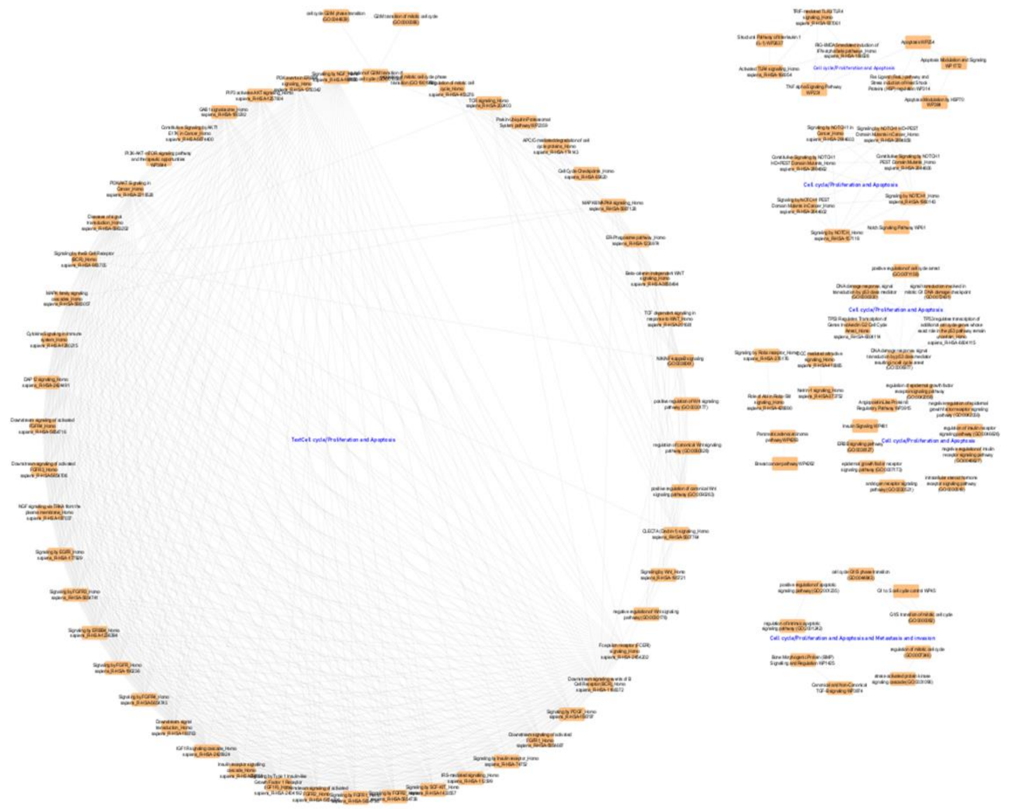

(c) Indole 3-Carbinol on ZR751

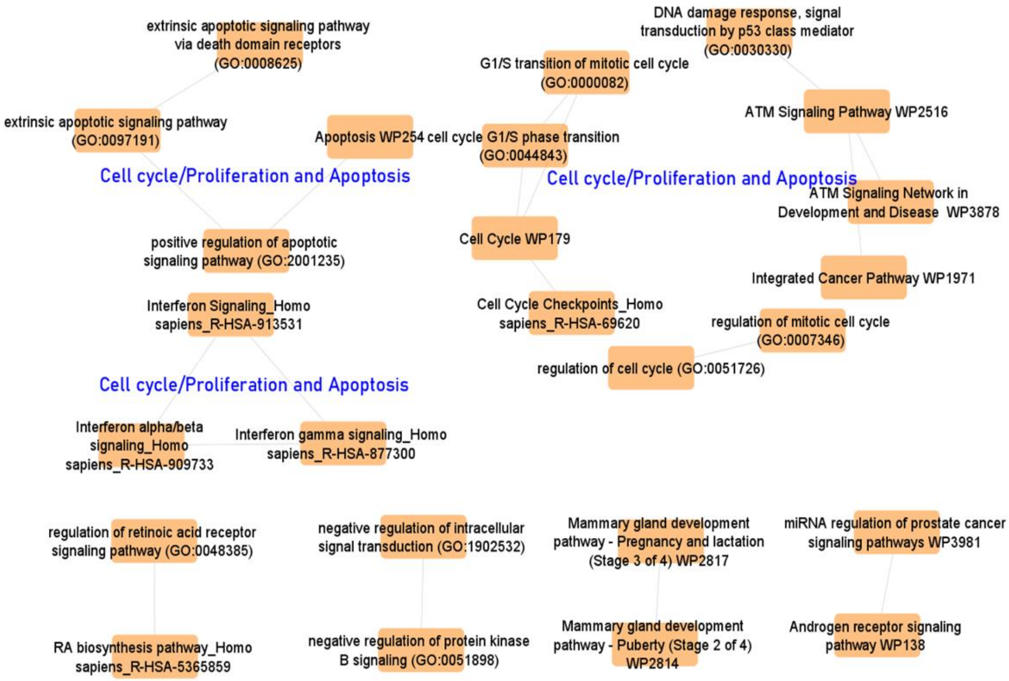

(d) Indole 3-Carbinol on T47D

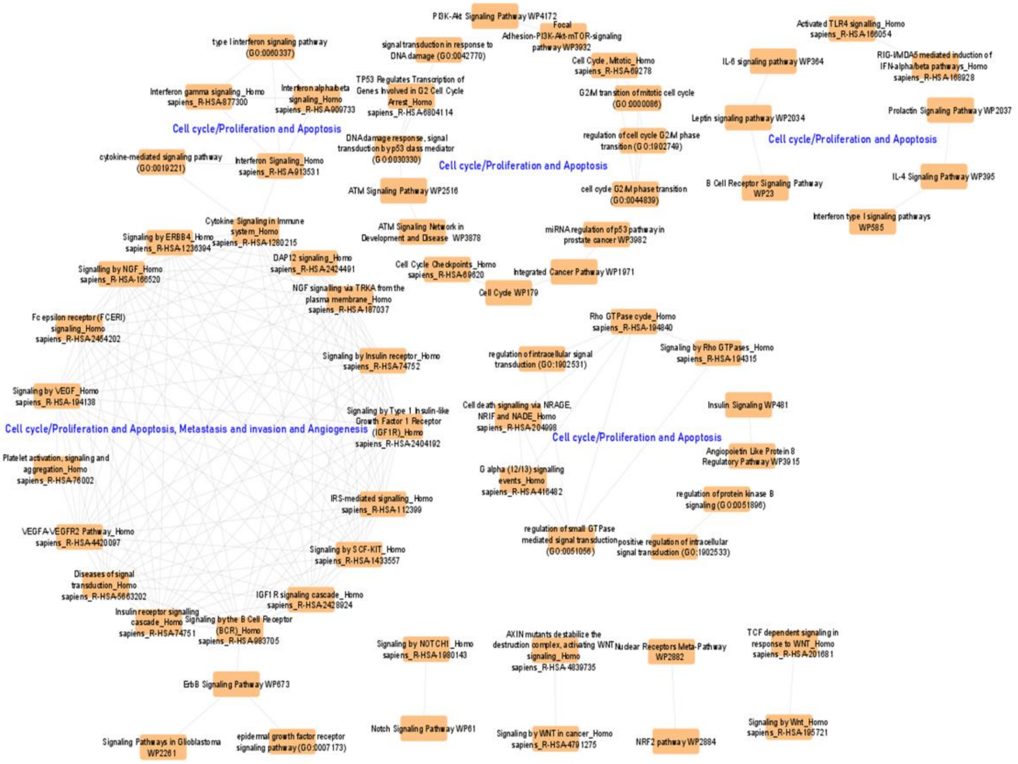

(e) Indole 3-Carbinol on MDA-MB-436

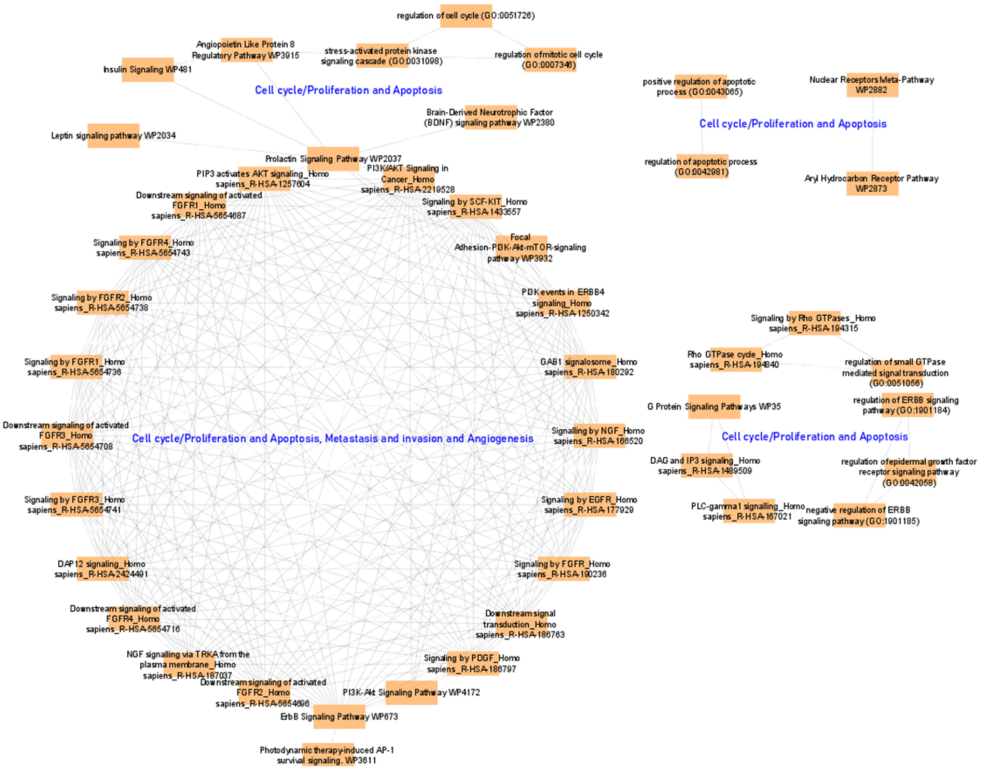

(f) Withaferin A on MCF-7

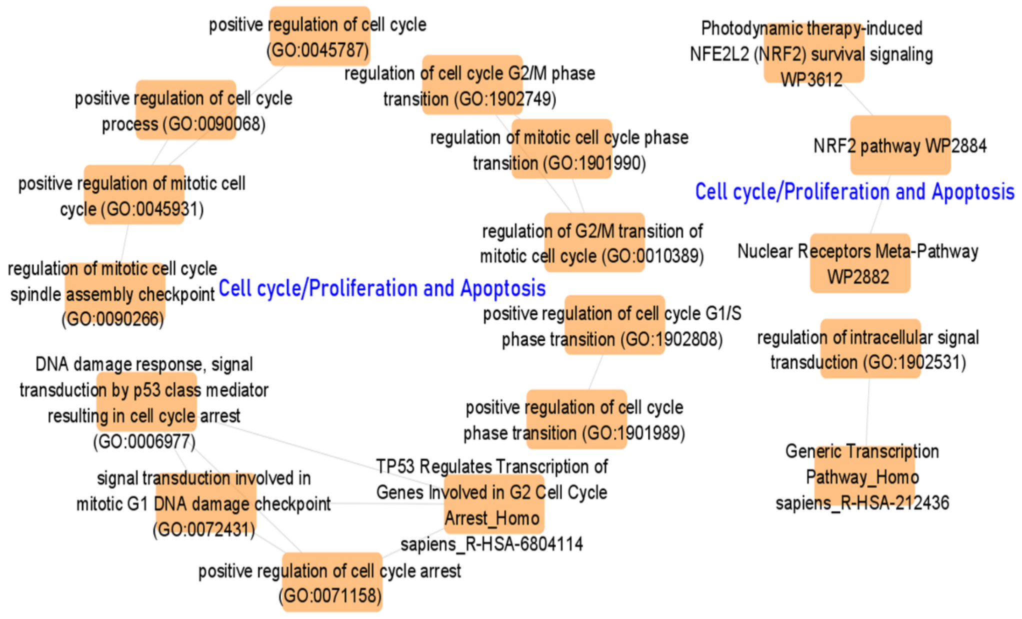

Supplement: Supplementary file 3 — Additional file 3: Supplementary Fig. 3. Pathway-pathway interaction networks based on shared enriched genes illustrating functional pathway cross-talk. a-f: represents networks of pathways targeted by CKI on MCF-7, I3C on MCF-7, I3C on MDA-MB-436, I3C on T47D, I3C on ZR751 and WA on MCF-7. CKI: Compound Kushen Injection, I3C: Indole 3-Carbinol, WA: Withaferin A. [file 12906_2021_3340_MOESM3_ESM.pdf]
