# Supplementary material for: A systems pharmacology approach based on oncogenic signalling pathways to determine the mechanisms of action of natural products in breast cancer from transcriptome data
Source: BMC Complement Med Ther. 2021 Jun 30;21:181. doi: 10.1186/s12906-021-03340-z (PMC8244196; doi:10.1186/s12906-021-03340-z)
Supplement: Supplementary file 4 — Additional file 4: Supplementary Table 1. Summary of the transcriptome datasets used and the molecular profiles of the cell lines. The columns Controls and Treatments list the number of samples in each case. (HER2+: human epidermal receptor 2 positive, ER+: Oestrogen receptor positive, TN: triple negative, AC: adenocarcinoma, IDC: invasive ductal carcinoma, MC: medullary carcinoma, Wt: wild type, Mut: Mutant, Del: deleted). [file 12906_2021_3340_MOESM4_ESM.pdf]

Table S1: Summary of the transcriptome datasets used and the molecular profiles of the cell lines. The columns Controls and Treatments list the number of samples in each case. (HER2+: human epidermal receptor 2 positive, LA: luminal A, TN: triple negative, AC: adenocarcinoma, IDC: invasive ductal carcinoma, MC: medullary carcinoma, Wt: wild type, Mut: Mutant, Del: deleted).

Drug	Platform	Cell line	Subtype	Pathology	BRCA1	P53	Controls	Treatments
Actein	Affymetrix Human Array	MDA-MB-453	HER2+	AC	Wt	Del	4	3
CKI	Illumina HiSeq 2500	MCF-7	LA	IDC	Wt	Wt	3	3
I3C	Illumina beadchip	MCF-7	LA	IDC	Wt	Wt	3	3
		T47D	LA	IDC	Wt	Mut	3	3
		ZR751	LA	IDC	Wt	Wt	3	3
		MDA-MB-231	TN	MC	Wt	Mut	3	3
		MDA-MB-157	TN	AC	Wt	Mut	3	3
		MDA-MB-436	TN	AC	Mut	Mut	3	3
WA	Illumina beadchip	MDA-MB-231	TN	MC	Wt	Mut	3	3
		MCF-7	LA	IDC	Wt	Wt	3	3
