# Supplementary material for: A systems pharmacology approach based on oncogenic signalling pathways to determine the mechanisms of action of natural products in breast cancer from transcriptome data
Source: BMC Complement Med Ther. 2021 Jun 30;21:181. doi: 10.1186/s12906-021-03340-z (PMC8244196; doi:10.1186/s12906-021-03340-z)
Supplement: Supplementary file 5 — Additional file 5: Supplementary Table 2. Summary of the differential expression analysis results. The number of differentially expressed genes under the respective plant-derived drugs/compounds are given in the table. DEG: Differentially expressed genes, FDR: False discovery rate, FC: Fold change. [file 12906_2021_3340_MOESM5_ESM.pdf]

Table S2: Summary of the differential expression analysis results. The number of differentially expressed genes under the respective plant-derived drugs/compounds are given in the table. DEG: Differentially expressed genes, FDR: False discovery rate, FC: Fold change

Drug/Compound	Dosage	Cell Line	DEGs	FDR cut-off	FC cut-off
Actein	40µg/ml	MDA-MB-453	520	0.01	2
CKI	2mg	MCF-7	1661	0.01	2
I3C	200µM	MCF-7	3115	0.005	2
		T47D	2462	0.005	2
		ZR751	2125	0.005	2
		MDA-MB-231	202	0.005	2
		MDA-MB-157	430	0.005	2
		MDA-MB-436	869	0.005	2
WA	700nM	MDA-MB-231	1614	0.005	2
		MCF-7	482	0.005	2
