# Supplementary material for: A systems pharmacology approach based on oncogenic signalling pathways to determine the mechanisms of action of natural products in breast cancer from transcriptome data
Source: BMC Complement Med Ther. 2021 Jun 30;21:181. doi: 10.1186/s12906-021-03340-z (PMC8244196; doi:10.1186/s12906-021-03340-z)
Supplement: Supplementary file 9 — Additional file 9: Supplementary Table 6. An example of Actein targeted oncogenesis processes illustrating the approach used in grouping the oncogenic signaling pathways into different cancer pathophysiological processes based on the pathways’ enriched genes. [file 12906_2021_3340_MOESM9_ESM.pdf]

Table S6: An example of the approach used in grouping the oncogenic signalling pathways into different cancer pathophysiological processes based on each pathway's enriched genes for MDA-MB-453 cell line under Actein treatment. The pathways highlighted here were derived from the enrichment analysis results of the whole subnetworks (**Supp Table 4**). Gene functions were derived from UniProt database. The reasons for considering each gene in arriving at a conclusion are given.

Oncogenic Signalling Pathway	Known biological role(s)	Gene	Gene Functions (UniProt)	Status of gene in decision	Targeted Oncogenesis Process
Intrinsic Pathway for Apoptosis_Homo sapiens_R-HSA-109606	(i) Cell death	TFDP1	(i) Binds DNA cooperatively with E2F family members whose products are involved in cell cycle regulation or in DNA replication (pubmed:8405995, pubmed:7739537).<br>(ii) The E2F1:DP complex appears to mediate both cell proliferation and apoptosis.	**Considered because of relation to cell apoptosis**	**Cell cycle/Proliferation and Apoptosis**
		E2F1	(i) Transcription activator that binds DNA cooperatively with DP proteins whose products are involved in cell cycle regulation or in DNA replication.<br>(ii) The DRTF1/E2F complex functions in the control of cell-cycle progression from G1 to S phase.<br>(iii) It can mediate both cell proliferation and TP53/p53-dependent apoptosis.	**Considered because of relation to cell apoptosis**	
		BID	(i) The major proteolytic product p15 BID allows the release of cytochrome c. Isoform 1, isoform 2 and isoform 4 induce ICE-like proteases and apoptosis. Isoform 3 does not induce apoptosis. Counters the protective effect of Bcl-2	**Considered because of relation to cell apoptotic genes**	
		BCL2L1	(i) Potent inhibitor of cell death.<br>(ii) Acts as a regulator of G2 checkpoint and progression to cytokinesis during mitosis.<br>(iii) May attenuate inflammation impairing NLRP1-inflammasome activation, hence CASP1 activation and IL1B release (pubmed:17418785).<br>(iv) Isoform Bcl-X(S) promotes apoptosis.	**Considered because of relation to cell apoptosis**	
Interferon Signaling_Homo sapiens_R-HSA-913531	(i) immune response to viral infection<br>(ii) antitumor immunity<br>(iii) cell growth inhibition	NUP93	(i) Regulates podocyte migration and proliferation through SMAD4 signalling (pubmed:26878725).	**Considered because of relation to cell proliferation**	**Cell cycle/Proliferation and Apoptosis**
		NDC1	(i) Component of the nuclear pore complex (NPC), which plays a key role in de novo assembly and insertion of NPC in the nuclear envelope.	**Considered because of relation to cell proliferation**	
		NUP205	(i) Plays a role in the nuclear pore complex (NPC) assembly and/or maintenance (pubmed:9348540).	**Not considered**	

		IFITM2	(i) IFN-induced antiviral protein which inhibits the entry of viruses to the host cell cytoplasm.<br>(ii) Induces cell cycle arrest and mediates apoptosis by caspase activation and in p53-independent manner.	**Considered because of relation to immune response**	
		NUP107	(i) Plays a role in the nuclear pore complex (NPC) assembly and/or maintenance (pubmed:12552102).	**Considered**	
		NUP188	(i) May function as a component of the nuclear pore complex (NPC).	**Not considered**	
		NUP155	(i) Essential for embryogenesis.<br>(ii) Involved both in binding and translocating proteins during nucleocytoplasmic transport.	**Not considered**	
		NUP85	(i) Essential component of the nuclear pore complex (NPC) that seems to be required for NPC assembly and maintenance (pubmed:12718872).<br>(ii) Involved in CCR2-mediated chemotaxis of monocytes and may link activated CCR2 to the phosphatidyl-inositol 3-kinase-Rac-lammellipodium protrusion cascade (pubmed:15995708).	**Considered because of relation to immune response**	
		OAS3	(i) Plays a critical role in cellular innate antiviral response<br>(ii) May also play a role in other cellular processes such as apoptosis, cell growth, differentiation and gene regulation.	**Considered because of relation to apoptosis**	
		TRIM14	(i) Plays a role in the innate immune defense against viruses<br>(ii) Facilitates the type I IFN response (pubmed:24379373)<br>(iii) Positively regulates the CGAS-induced type I interferon signalling pathway (pubmed:27666593)	**Considered because of relation to immune response**	
		NUP160	(i) Involved in poly(A)+ RNA transport.	**Not considered**	
		PIAS1	(i) Functions as an E3-type small ubiquitin-like modifier (SUMO) ligase, stabilizing the interaction between UBE2I and the substrate, and as a SUMO-tethering factor<br>(ii) Plays a crucial role as a transcriptional coregulation in the STAT pathway, the p53 pathway and the steroid hormone signalling pathway.	**Considered because of relation to cell proliferation and death pathways**	
PI3K-AKT-mTOR signaling pathway and therapeutic opportunities WP3844	(i) apoptosis<br>(ii) autophagy<br>(iii) metastasis<br>(iv) cell growth	RB1CC1	(i) Involved in autophagy (pubmed:21775823).<br>(ii) Involved in repair of DNA damage caused by ionizing radiation, which subsequently improves cell survival by decreasing apoptosis (By similarity).<br>(iii) Plays a role as a modulator of TGF-beta-signalling.	**Considered because of relation to autophagic cell death**	**Cell cycle/Proliferation and Apoptosis**
		EIF4EBP1	(i) Mediates the regulation of protein translation by hormones, growth factors and other stimuli that signal through the MAP kinase and mtorc1 pathways	**Considered because of relation to cell growth and proliferation**	

		GRB10	(i) Binds to, and suppress signals from, activated receptors tyrosine kinases, including the insulin (INSR) and insulin-like growth factor (IGF1R) receptors.<br>(ii) Negatively regulates Wnt signalling.<br>(iii) Positive regulator of the KDR/VEGFR-2 signalling pathway	**Considered because of relation to cell growth and proliferation**	
		PTEN	(i) Tumor suppressor.<br>(ii) Antagonizes the PI3K-AKT/PKB signalling pathway thereby modulating cell cycle progression and cell survival.<br>(iii) The nuclear monoubiquitinated form possesses greater apoptotic potential, whereas the cytoplasmic nonubiquitinated form induces less tumor suppressive ability.	**Considered because of relation to cell proliferation and apoptosis**	
		ULK1	(i) Involved in autophagy in response to starvation. (pubmed:25040165).	**Considered because of relation to autophagic cell death**	
NRF2 pathway WP2884	(i) detoxification and metabolism of xenobiotics<br>(ii) cell differentiation and apoptosis<br>(iii) cell proliferation	ABCC3	(i) Act as an inducible transporter in the biliary and intestinal excretion of organic anions.	**Considered because of relation to cell proliferation**	**Cell cycle/Proliferation and Apoptosis**
		GCLC	(i) This protein is involved in step **1** of the subpathway that synthesizes glutathione from L-cysteine and L-glutamate	**Considered because of relation to cell proliferation**	
		SLC2A10	(i) Facilitative glucose transporter required for the development of the cardiovascular system	**Not considered**	
		TXNRD1	(i) Isoform 5 also mediates cell death induced by a combination of interferon-beta and retinoic acid	**Considered because of relation to immune mediated cell death**	
		MAFG	(i) Serve as transcriptional activators by dimerizing with other (usually larger) basic-zipper proteins, such as NFE2, NFE2L1 and NFE2L2, and recruiting them to specific DNA-binding sites (pubmed:8932385, pubmed:9421508, pubmed:11154691).<br>(ii) Small Maf proteins heterodimerize with Fos and may act as competitive repressors of the NFE2L2 transcription factor (pubmed:11154691).<br>(iii) May be involved in signal transduction of extracellular H+ (By similarity)	**Not considered**	
		FTH1	(i) Stores iron in a soluble, non-toxic, readily available form. Important for iron homeostasis.<br>(ii) Also plays a role in delivery of iron to cells.	**Not considered**	
		HMOX1	(i) Exhibits cytoprotective effects since excess of free heme sensitizes cells to undergo apoptosis.	**Considered because of relation to apoptosis**	

		GCLM	(i) This protein is involved in step **1** of the subpathway that synthesizes glutathione from L-cysteine and L-glutamate.	**Not considered**	
		FGF13	(i) Is involved in both polymerization and stabilization of microtubules.	**Not considered**	
		SQSTM1	(i) Autophagy receptor required for selective macroautophagy (aggrephagy) (pubmed:16286508, pubmed:20168092, pubmed:24128730, pubmed:28404643, pubmed:22622177).<br>(ii) May be involved in cell differentiation, apoptosis, immune response and regulation of K+ channels.<br>(iii) Acts as an activator of the NFE2L2/NRF2 pathway (pubmed:20452972, pubmed:28380357).	**Considered because of relation to apoptosis, immune response and autophagy**	
		SLC39A14	(i) Broad-scope metal ion transporter with a preference for zinc uptake (pubmed:29621230).	**Not considered**	
		FTL	(i) Stores iron in a soluble, non-toxic, readily available form.<br>(ii) Plays a role in delivery of iron to cells.	**Not considered**	
TGF-beta Signaling Pathway WP366	(i) cancer progression<br>(ii) invassion<br>(iii) metastasis<br>(iv) cell growth and survival<br>(v) cell cycle arrest<br>(vi) apoptosis<br>(vii) angiogenesis<br>(viii) inflammation	TGIF1	(i) Active transcriptional corepressor of SMAD2. Links the nodal signalling pathway to the bifurcation of the forebrain and the establishment of ventral midline structures.	**Considered because of relation to cell proliferation**	**Cell cycle/Proliferation and Apoptosis**
		APP	(i) Functions on the surface of neurons relevant to neurite growth, neuronal adhesion and axonogenesis.<br>(ii) Couples to apoptosis-inducing pathways such as those mediated by g(o) and jip.	**Considered because of relation to apoptosis**	
		CDKN1A	(i) Involved in p53/TP53 mediated inhibition of cellular proliferation in response to DNA damage.<br>(ii) Binds to and inhibits cyclin-dependent kinase activity and blocking cell cycle progression. (pubmed:11595739)	**Considered because of relation to cell proliferation**	
		ITCH	(i) Acts as an E3 ubiquitin-protein ligase (pubmed:14602072,).<br>(ii) Mediates the antiapoptotic activity of epidermal growth factor through the ubiquitination and proteasomal degradation of p15 BID (pubmed:20392206).	**Considered because of relation to apoptosis**	
		KLF6	(i) Could play a role in B-cell growth and development	**Considered because of relation to immune response**	
		NEDD4L	(i) E3 ubiquitin-protein ligase<br>(ii) Inhibits TGF-beta signalling by triggering SMAD2 and TGFBR1 ubiquitination and proteasome-dependent degradation.<br>(iii) Involved in the regulation of TOR signalling (pubmed:27694961).	**Considered because of relation to cell proliferation**	
		ATF3	(i) This protein binds the camp response element (CRE) (consensus: 5'-GTGACGT[AC][AG]-3'), a sequence present in many viral and cellular promoters. Represses transcription from promoters with ATF sites.	**Considered because of relation to apoptosis**	
